# Do Conventional Meat-Purchase Motivations Predict Acceptance of Cultured Meat? A National Study Among Polish Consumers

**DOI:** 10.3390/foods15040746

**Published:** 2026-02-18

**Authors:** Anna M. Kaczmarek

**Affiliations:** Department of Food Quality and Safety Management, Faculty of Food Science and Nutrition, Poznań University of Life Sciences, Wojska Polskiego 31, 60-637 Poznań, Poland; anna.kaczmarek@up.poznan.pl

**Keywords:** cultured meat, consumer acceptance, alternative proteins, behavioural segmentation, psychographic segmentation, technological risk perception, food technology neophobia, sustainability attitudes, cellular agriculture, Poland

## Abstract

Cultured meat is increasingly considered a potential complement to conventional meat, yet the determinants of its acceptance remain unclear. This study examined whether motivations underlying conventional meat purchasing are associated with attitudes and behavioural intentions toward cultured meat among adult Polish meat eaters (*n* = 425). A cross-sectional online ssurvey assessed attitudes, perceived risks, general acceptance, behavioural intentions and socio-demographic characteristics. Overall attitudes and acceptance were moderately positive, while concerns related to technological risk and naturalness persisted. Four psychographic segments were identified, with cautious optimists (35.9%) and concerned ambivalents (33.3%) representing the largest groups. Associations between conventional meat-purchase motivations and attitudes toward cultured meat were statistically significant but modest, with ethical and environmental motives showing weak positive associations and sensory-oriented motives showing weak negative ones. The correspondence between segmentation based on conventional meat motivations and that based on cultured-meat orientations was limited, indicating only partial structural overlap. Younger, urban and higher-educated respondents were disproportionately represented in the more favourable segments, and prior familiarity increased the likelihood of positive attitudes. Overall, the findings indicate that motivations for purchasing conventional meat explain only a limited share of variability in cultured meat acceptance. Factors related to familiarity, perceived technological characteristics and broader psychosocial orientations appear more influential and should be explored further in future research.

## 1. Introduction

It is evident that global demand for animal protein has undergone a significant escalation over the past six decades, a phenomenon that can be attributed to a combination of population growth, rising incomes, and rapid urbanisation [1,2]. As demonstrated by Fu et al. (2023) [3] global meat consumption has increased more than fourfold since 1961, placing substantial pressure on ecosystems, natural resources, and climate stability. This trajectory, when viewed in conjunction with mounting public concern regarding animal welfare, zoonotic disease risks, and the sustainability of conventional livestock systems, has catalysed global interest in alternative protein sources [4]. In this rapidly evolving landscape, cultured meat has emerged as one of the most technologically advanced and potentially transformative solutions [5,6]. Cultured meat is one among multiple emerging pathways being explored in response to the sustainability challenges of current protein systems. These pathways include plant-based proteins, insect-based products, precision fermentation-derived ingredients [6], as well as non-technological approaches such as dietary protein reduction, sustainable livestock intensification, and agroecological transformation [7,8]. The notion of a ‘protein transition’ remains contentious, encompassing a range of narratives that are at times incongruent. These narratives encompass the equilibrium between supply-side innovations (e.g., alternative proteins) and demand-side interventions (e.g., dietary shifts), the role of technological versus ecological solutions, and the appropriate scope for systemic versus incremental change [9,10]. The pathways diverge significantly in terms of technological readiness, consumer acceptance, environmental impact, and congruence with prevailing food systems [11]. Recognizing this pluralism, cultured meat should be understood not as a singular solution but as one component within a broader portfolio of strategies aimed at rebalancing protein production and consumption. A comprehensive understanding of consumer motivations regarding cultured meat is therefore essential for informed participation in debates about which combinations of transition pathways may be most viable, equitable, and culturally appropriate in different regional and regulatory contexts.

Cultured meat (CM), also referred to as cultivated, cell-based or lab-grown meat, is produced by proliferating animal cells ex vivo in controlled bioreactor environments, thereby aimed at replicating the biological and nutritional characteristics of conventional meat without the need to raise or slaughter animals [12,13]. In recent years, there has been a substantial acceleration in the progress of cellular agriculture. Documented advances in the field, as outlined by Lee (2025), Xie (2025), Tan (2025) and Olenic (2025) [14,15,16,17], demonstrate that advancements in cell-line development, edible scaffolds, bioprocess optimisation and mechanobiology are contributing to the development of more robust and scalable production systems. These developments are supported by strategic evaluations of industrial feasibility and regulatory frameworks, such as those provided by Zandonadi (2025) [18] and Sun (2025) [19], which highlight both the promise and remaining constraints associated with large-scale bioreactor-based production.

The potential benefits of cultured meat have been extensively articulated. In comparison with traditional livestock farming, cultured meat has the potential to significantly reduce land use, water consumption and greenhouse gas emissions [20], whilst eliminating the necessity for antibiotics and reducing exposure to zoonotic pathogens [21,22]. Furthermore, the advent of cultured meat systems has engendered unparalleled command over nutritional composition and product safety, thereby facilitating the conception of meat with bespoke health profiles [23]. Nevertheless, despite its substantial promise, cultured meat faces considerable technological, sensory and economic challenges. As posited by Bourdez et al. (2022) [24] and Xie (2025) [15], the factors impeding the rapid commercial adoption of meat produced through biotechnology include high production costs, uncertainties related to large-scale bioprocessing, and difficulties in replicating the sensory properties of traditionally produced meat. Concerns regarding the naturalness and safety of highly engineered foods further complicate public acceptance.

Recent analyses emphasize that technological innovation and policy instruments alone are insufficient to drive a meaningful protein transition unless consumers are willing to adopt novel protein sources. As highlighted by Bryant et al. (2024) [25], even well-designed policy levers aimed at reducing conventional meat production and consumption may remain ineffective without adequate levels of public acceptance and behavioural readiness. This underscores that consumer attitudes are not a peripheral consideration but a central determinant shaping the feasibility of cellular agriculture and, more broadly, the future of sustainable protein systems. Understanding how individuals evaluate, perceive, and emotionally respond to cultured meat is therefore essential for assessing its market potential and for informing evidence-based communication and policy strategies.

In recent years, there has been a notable increase in research activity concerning consumer attitudes towards cultured meat, particularly during the 2024–2025 period. A multitude of international studies have emphasised that consumer openness varies significantly across a variety of cultural, socio-demographic and psychological contexts. For instance, Rodríguez Escobar et al. (2025) [26] identified significant variations in acceptance between Belgium, Chile and China, while Stanco et al. (2025) [27] documented intricate patterns of ambivalence and curiosity among Italian consumers. Further research from Greece [28], Romania [29], Brazil [30] and the UAE [31] lends further support to the view that acceptance is shaped by distinct cultural norms, dietary traditions and underlying trust in food technologies. It is important to note that the extant evidence base for Poland remains limited, with only a small number of earlier studies examining general attitudes towards cultured meat in the Polish population [32] and none providing an integrated analysis of awareness, attitudinal structures, behavioural intentions or segmentation. This underscores the necessity for the present study.

The present study operationalises four core psychological constructs that have been identified in systematic reviews as central to understanding consumer responses to cellular agriculture technologies [5,13]. Drawing on established attitude–behaviour frameworks and food technology acceptance research, each construct is defined below with reference to its theoretical foundations and relevance to the research question.

Attitudes toward cultured meat (ATT_CM) are defined as overall evaluative dispositions reflecting the degree to which an individual views cultured meat favourably or unfavourably [33]. Following the tripartite model of attitudes, this construct integrates cognitive beliefs about product attributes (e.g., perceived healthiness, environmental benefits), affective responses to the technology (e.g., feelings of curiosity, disgust, or enthusiasm), and behavioural predispositions (e.g., willingness to consider the product). In the domain of food technology acceptance research, attitudes have been consistently identified as the most proximal and powerful predictor of behavioural intentions and subsequent adoption [34]. The theory of planned behaviour posits that attitudes, alongside subjective norms and perceived behavioural control, determine behavioural intentions, which in turn predict actual behaviour [35]. The inclusion of ATT_CM is therefore justified by its theoretical centrality in attitude–behaviour models and its demonstrated predictive validity across diverse food innovation contexts, including novel proteins [36,37].

Behavioural intentions toward cultured meat (INT_CM) are defined as the subjective probability that an individual will engage in a specific behaviour—in this case, trying, purchasing, or recommending cultured meat [35]. Intentions represent the motivational precursors to actual behaviour and capture the degree of effort an individual is willing to exert to perform the behaviour. In accordance with the theory of planned behaviour, intentions mediate the relationship between attitudes and behaviour, making them essential for predicting market readiness. Intentions provide a more action-relevant outcome measure than attitudes alone [38] and are considered essential for assessing the size and characteristics of potential early-adopter segments [39]. Meta-analytic evidence confirms that behavioural intentions are the strongest predictor of actual food choice behaviour, particularly for novel or unfamiliar products where direct experience is limited.

Technological risk and naturalness concern (TRNC) encapsulates perceptions of artificiality, apprehension related to unfamiliarity, and perceived risks associated with technologically mediated food production [40]. This construct captures the psychological discomfort that arises when consumers perceive food products as “unnatural,” overly processed, or produced through unfamiliar biotechnological methods. The construct is informed by the extensive literature on food technology neophobia, which demonstrates that consumers vary systematically in their discomfort with novel food technologies [41]. The Food Technology Neophobia Scale and related instruments have consistently shown that perceptions of unnaturalness function as among the strongest psychological barriers to acceptance of novel food technologies, including cultured meat [42]. Individuals scoring highly on TRNC tend to perceive cultured meat as unnatural, unsafe, or excessively complex, which functions as a cognitive and affective barrier to acceptance regardless of the objective attributes of the technology.

General acceptance of cultured meat (GACM) is defined as a broader evaluative construct encompassing three dimensions: (1) the perceived societal need for the technology, (2) its perceived relative advantage over alternative protein sources, and (3) alignment with personal values and standards [39,43]. This construct extends beyond individual attitudes to incorporate normative and value-based dimensions of acceptance. Drawing on the diffusion of innovation framework proposed by Rogers (2003) [39], GACM captures the extent to which cultured meat is perceived as compatible with existing value systems and superior to available alternatives—factors that predict adoption beyond the immediate personal evaluation stage. These normative and comparative evaluations have been identified as significant drivers in sustainability-oriented food transitions, where consumers assess not only personal benefits but also broader societal implications [43]. The inclusion of GACM allows the study to capture acceptance dimensions that transcend individual hedonic or utilitarian evaluations.

Collectively, these four constructs—ATT_CM, INT_CM, TRNC, and GACM—constitute a concise yet comprehensive framework that is fully aligned with current international research on consumer responses to novel protein technologies. The constructs capture complementary dimensions of consumer response: general evaluations (ATT_CM), behavioural readiness (INT_CM), psychological barriers (TRNC), and broader normative acceptance (GACM). This multi-dimensional operationalisation enables a nuanced understanding of how consumers position cultured meat within their food choice frameworks.

The central research question of the present study seeks to answer whether established motivations for purchasing conventional meat can predict consumer responses to cultured meat, as operationalised through the constructs defined above. Drawing on theoretical perspectives from consumer psychology, food-choice research, and technology adoption, four hypotheses are developed below.

Ethical and environmental motivations for food choice are defined as reflective, value-driven decision orientations rooted in concerns about animal welfare, environmental sustainability, and the broader societal impacts of food production [44,45]. These motivations represent higher-order cognitive evaluations that extend beyond immediate hedonic gratification to encompass moral and normative considerations regarding the consequences of dietary choices. In the context of conventional meat purchasing, ethical motivations manifest as preferences for products perceived as more humane, sustainable, or aligned with personal values regarding animal welfare and environmental stewardship [46,47].

The relationship between ethical motivations for conventional meat and attitudes toward cultured meat (ATT_CM) can be understood through two complementary theoretical frameworks. First, dual-attitude models posit that food-choice behaviour is shaped jointly by reflective evaluations and automatic affective responses [48,49]. Consumers who prioritise ethical and environmental considerations engage in deliberative processing that weighs broader consequences against immediate preferences—a cognitive orientation that should facilitate positive evaluation of technologies promising ethical benefits. Second, the moralised eating framework suggests that concerns about animal welfare and environmental protection operate as stable moral values that generalise across food domains [46,50]. De Backer and Hudders (2015) [46] demonstrated that individuals motivated by animal welfare and environmental ethics exhibit distinct dietary patterns and a greater willingness to adopt animal-friendly alternatives. Because cultured meat is explicitly positioned as addressing animal welfare concerns (no slaughter required) and environmental impacts (reduced land use, emissions), consumers with strong ethical motivations should perceive value alignment and express more favourable attitudes.

While value generalisation theory predicts positive transfer from ethical motivations to cultured meat attitudes, competing perspectives suggest this transfer may be incomplete. Cultured meat represents a technological intervention that may trigger ambivalence even among ethically-motivated consumers who harbour reservations about “unnatural” food production methods [40,51]. The technological-neophobia literature indicates that perceptions of artificiality can generate negative affective responses (captured by TRNC) that override cognitive endorsement of a product’s ethical credentials [42]. Furthermore, some ethically-oriented consumers may prefer demand-side solutions (e.g., plant-based diets) over supply-side technological fixes, viewing cultured meat as an incomplete response to systemic problems of industrial animal agriculture [52,53]. The extent to which ethical motivations predict ATT_CM is therefore an empirical question. The present study proposes a theory that is predicated on the value generalisation hypothesis and empirical evidence that ethical orientations predict openness to sustainability-oriented food innovations [37,46]. The theory is as follows:

**H1:**  *Ethical and environmental motivations for purchasing conventional meat are positively associated with more favourable attitudes toward cultured meat.*

This association is consistent with the findings of recent meta-analytic studies that identified perceived ethicality as the strongest positive predictor of willingness to consume cultured meat across 48 empirical studies [54]. Notably, younger consumers are particularly motivated by considerations of animal welfare and environmental sustainability [55].

Sensory-oriented motivations for food choice are defined as preferences driven by immediate hedonic attributes including taste, texture, appearance, and smell [45]. These motivations reflect experiential, affective processing that prioritises sensory pleasure, familiarity, and immediate gratification over abstract considerations such as health, ethics, or environmental impact. In the context of conventional meat purchasing, sensory motivations manifest as strong preferences for specific taste profiles, textures, and eating experiences that are deeply embedded in habitual consumption patterns [47]. As posited by Malila et al., [56] the technological challenges associated with replicating the sensory properties of conventional meat—including taste, texture, and appearance—represent fundamental barriers to consumer acceptance that extend beyond attitudinal predispositions.

The theoretical relevance of sensory motivations derives from the technological neophobia framework, which posits that individuals with strong hedonic preferences and attachment to familiar food experiences exhibit heightened discomfort with products perceived as artificial, novel, or technologically mediated [41,42]. Consumers who prioritise sensory attributes when purchasing conventional meat are likely to evaluate cultured meat primarily through an experiential lens, where unfamiliarity with the production process and uncertainty regarding sensory outcomes function as barriers. This mechanism should affect not only attitudes (ATT_CM) but also behavioural intentions (INT_CM), as sensory-oriented consumers may be unwilling to try a product with uncertain sensory properties and general acceptance (GACM), in turn because such consumers may not perceive cultured meat as a satisfactory alternative to conventional meat.

While the technological neophobia perspective predicts negative associations, cultured meat producers explicitly aim to replicate the sensory properties of conventional meat [15]. If successful sensory replication is achieved and effectively communicated, sensory-oriented individuals might ultimately represent a receptive market segment. Nevertheless, at the present stage of market development, characterised by restricted product availability, ongoing sensory uncertainty, and an absence of direct tasting experience, negative associations are more theoretically congruent with the neophobia framework. Furthermore, elevated TRNC scores among sensory-oriented consumers have the potential to amplify resistance by activating concerns regarding “unnatural” production methods. In accordance with the technological neophobia hypothesis, as well as the evidence that sensory-focused consumers exhibit greater scepticism towards novel food technologies [34,51], he following proposal is put forward:

**H2:**  *Sensory-oriented motivations for purchasing conventional meat are negatively associated with attitudes toward cultured meat and with lower acceptance and behavioural intentions toward cultured meat.*

This expectation is corroborated by a recent scoping review which demonstrated that taste and texture constitute fundamental determinants of meat purchasing decisions, with sensory uncertainty functioning as a primary barrier to cultured meat adoption among consumers who prioritise hedonic food attributes [57,58].

Consumer segmentation refers to the classification of individuals into homogeneous subgroups based on shared characteristics [34]. In the present study, behavioural segmentation refers to clusters derived from conventional meat-purchase motivations (the relative importance of sensory, ethical, health-related, and economic attributes), while psychographic segmentation refers to clusters derived from the four cultured meat constructs: ATT_CM, INT_CM, TRNC, and GACM. If motivations for purchasing conventional meat systematically relate to cultured meat responses (as proposed in H1 and H2), then consumer segments derived from these motivations should exhibit meaningful correspondence with psychographic clusters based on the four outcome constructs. This structural hypothesis tests whether individual-level associations aggregate into coherent segment-level patterns. Theoretical consistency would predict that ethically-oriented meat purchasers cluster with cultured-meat enthusiasts (high ATT_CM, high INT_CM, low TRNC, high GACM), while sensory-focused purchasers align with more sceptical segments.

While motivational consistency predicts substantial overlap, cultured meat acceptance may be shaped by factors not captured in meat-purchase motivations. These include familiarity with cellular agriculture, trust in regulatory institutions, and affective responses to biotechnology [13,59], factors that are captured by TRNC but not by conventional meat-choice heuristics. The literature on alternative proteins suggests that acceptance patterns do not always align across categories; consumers may embrace plant-based alternatives while rejecting cultured meat [60]. The degree of structural correspondence is therefore an empirical question. Recent cross-cultural research employing the values–attitudes–behaviour model demonstrates that meat attachment operates as a mediator between consumer characteristics and cultured meat acceptance, suggesting meaningful structural correspondence between conventional meat orientation and psychographic response profiles [26]. It is proposed that food-choice patterns provide partial but meaningful anchors for understanding responses to novel technologies [61].

**H3:**  *Segments derived from conventional meat-purchasing motivations demonstrate statistically significant overlap with clusters based on cultured-meat attitudes and behavioural intentions.*

Demographic variation refers to systematic differences in psychographic segment composition across sociodemographic groups defined by age, education, urbanisation, and other structural characteristics. The diffusion of innovation framework [39] posits that early adopters of disruptive technologies exhibit specific demographic profiles: younger age, higher education, greater cosmopolitanism, and higher tolerance for uncertainty. These demographic factors should moderate the relationship between meat-purchase motivations and cultured meat acceptance: younger, urban, higher-educated consumers with ethical motivations should be disproportionately represented in favourable psychographic segments (high ATT_CM, high INT_CM, high GACM, low TRNC). Empirical evidence from international studies [36,62,63] consistently documents higher acceptance among these demographic groups. Recent large-scale segmentation research has confirmed the existence of systematic generational variation in receptivity to cultured meat. The research indicates that gen Z (88%) and millennials (85%) demonstrate substantially higher levels of openness than gen X (77%) and baby boomers (72%). The study also found consistent positive associations between educational attainment and acceptance across multiple cultural contexts [55,64].

Alternative perspectives suggest demographic effects may be attenuated. Digital information channels may reduce knowledge gaps between demographic groups [13]. Ethical concerns are not confined to younger consumers and may emerge across categories in response to media coverage [46]. Cultural factors may interact with demographics in complex ways [26,65]. The magnitude of demographic variation in segment composition therefore warrants empirical examination. In light of the diffusion of innovation theory and the existence of consistent empirical evidence [36,37,66] the following proposal is hereby put forward:

**H4:**  *The alignment between ethical meat-purchase motivations and positive attitudes toward cultured meat varies across demographic groups, such that younger, urban, and higher-educated consumers are disproportionately represented in more favourable psychographic segments.*

The hypotheses presented above establish a theoretically grounded framework linking conventional meat-purchase motivations to cultured meat acceptance. The four outcome constructs—ATT_CM, INT_CM, TRNC, and GACM—capture complementary dimensions of consumer response, which include general evaluations, behavioural readiness, psychological barriers, and broader normative acceptance. The present study aims to explore the individual-level associations between specific motivational orientations (ethical/environmental vs. sensory) and outcome constructs. The present study extends the analysis to the segment level, with the aim of testing the hypothesis that behavioural clusters derived from meat-purchase motivations correspond structurally to psychographic clusters based on the four constructs of cultured meat. The H4 model incorporates demographic heterogeneity, the purpose of which is to examine whether the proposed associations vary systematically across sociodemographic groups. The combination of these hypotheses provides a comprehensive analytical framework for understanding whether established food-choice patterns predict openness to emerging protein technologies, and, if so, under what conditions this would occur.

The present study’s focus on cultured meat does not imply endorsement of a singular or technologically deterministic pathway for protein system transformation. As emphasised in recent reviews [7,52], sustainable protein futures are inherently pluralistic, encompassing diverse strategies such as alternative proteins, regenerative agriculture, dietary shifts, and policy interventions. The decision to examine cultured meat is indicative of its status as an emerging and under-explored innovation within the Polish context, rather than a normative claim regarding its superiority. Research on consumer acceptance of individual pathways remains valuable, provided it is situated within this broader, contested landscape of food system change, and has established a direct bridge between the behavioural foundations of traditional meat consumption and the emergent acceptance patterns for cultured meat.

## 2. Materials and Methods

### 2.1. Study Design and Sample

The present study was conceived with the objective of examining the determinants of perceptions, attitudes, acceptance and behavioural intentions towards cultured meat among adult Polish consumers who currently consume meat. A cross-sectional online survey was conducted in Poland between May 2024 and June 2025 to assess consumer awareness, attitudes, perceived risks, and acceptance of cultured meat. The distribution of the questionnaire was conducted through SurvGo, utilizing university mailing lists and social media channels. A total of 483 complete responses were collected for the study. The analytical focus on meat eaters reflects the positioning of cultured meat as a substitute for conventional meat, and therefore the primary potential market is composed of individuals who routinely include meat in their diets. The original dataset comprised all respondents who completed the questionnaire, yielding a total of 483 fully completed responses. However, only those who declared that they consume meat with some regularity were retained for further analysis. Individuals who reported never or very rarely consuming meat were excluded to ensure conceptual coherence between the target market of cultured meat and the analytical sample. The final dataset employed in all statistical analyses comprised 425 adult Polish meat eaters.

The questionnaire comprised thematic blocks covering the following areas: Awareness of cultured meat (Q1–Q3), meat consumption and purchasing motives (Q4–Q7), attitudes toward cultured meat (Q10–Q16), perceived risks and unnaturalness (Q18.1–Q18.5), behavioural intentions related to cultured meat (Q17.1–Q17.5) and demographic characteristics (Q22–Q28). After the initial awareness questions, respondents received a short informational note describing cultured meat and its potential benefits (Supplementary Table S1), which was provided to ensure a minimum common understanding of the concept. The complete item wording and the structures of the measurement scales are presented in the Supplementary Material (Table S2).

The sociodemographic variables were employed to characterise the sample and to examine the sociodemographic determinants of the awareness of cultured meat. Finally, the variables were used to profile the consumer segments identified through clustering.

### 2.2. Psychometric Measures, Construct Operationalisation, and Validation

The operationalisation of several psychometric constructs was achieved through the utilisation of multi-item Likert-type scales. All scoring procedures were performed using listwise deletion within each construct-specific analysis, unless stated otherwise.

Attitudes toward cultured meat (ATT_CM; Q10–Q16) were assessed using a seven-item Likert scale capturing general evaluative dispositions. One item (Q13), which expressed a feeling of disgust toward cultured meat, was reverse-coded to ensure directional consistency before computing the composite score. Behavioural intentions (INT_CM; Q17.1–Q17.5) were measured using a series of five items reflecting willingness to try, purchase and recommend cultured meat. The technological risk and naturalness concern (TRNC; Q18.1–Q18.5) component was designed to capture perceptions of artificiality, novelty-related apprehension, and perceived risks. As higher original scores represented lower concern, items were reverse-coded to construct an index reflecting neophobia, such that higher scores indicated stronger apprehension. The general acceptance of cultured meat (GACM; Q19–Q21) scale was developed as a three-item measure designed to capture three distinct concepts. The integration of the three items comprising the GACM scale into a composite index was theoretically justified, despite the fact that these items reflect different evaluative domains. These domains are societal relevance, perceived technological superiority, and alignment with personal values. In the context of emerging food technologies, these evaluative dimensions are often cognitively integrated in consumers’ general acceptance judgments. Firstly, the index gauges the perceived societal need for cultured meat. Secondly, it assesses the relative advantage of cultured meat over other meat alternatives. Thirdly, it determines the degree to which cultured meat aligns with respondents’ personal standards and values. The computation of composite scores was conducted as arithmetic means of the corresponding items, under the condition that a sufficient number of item responses were available. The measurement instruments utilized in the present study were systematically adapted from validated psychometric scales to ensure cross-study comparability. The attitude (ATT) items (Q10–Q16) and behavioural intention (INT) items (Q17.1–Q17.5) were operationalised based on the frameworks developed by Wilks and Phillips (2017) and Bryant et al. (2019) [36,37], which are considered benchmark instruments in cultured meat research. Dimensions of perceived risk and unnaturalness (TRNC) were derived from the food technology neophobia scale (FTNS) [41] and naturalness perception measures established by Siegrist and Hartmann [42]. Conventional meat-purchase motivations (Q7.1–Q7.8) were based on the meat attachment questionnaire [47] and the classic food choice questionnaire [45]. All items underwent a rigorous translation and back-translation procedure to verify structural integrity within the Polish sample.

Internal consistency of each multi-item construct was evaluated using Cronbach’s alpha (α) and McDonald’s omega (Ω). Cronbach’s alpha is a measure of the average inter-item correlation within a scale. In contrast, McDonald’s omega is a more robust reliability estimate under a congeneric measurement model. This makes it particularly informative for constructs that include reverse-coded items. For each scale, alpha, omega, the number of valid cases, the number of items, and the mean and standard deviation of the composite score were computed. All reliability analyses were conducted in Python (version 3.13.3) using custom functions that relied on the use of a covariance-based eigen-decomposition for the calculation of omega. Complete item wordings and response formats are provided in Table S2 (Supplementary Materials). Table S3 presents construct-item mappings, reliability coefficients, and literature sources for all psychometric scales. Awareness items (Q1–Q3) served as covariates in logistic regression analyses predicting segment membership. Meat-purchase motivation items (Q7.1–Q7.8), adapted from Steptoe et al. (1995) [45], were used as clustering variables in K-means analysis to derive motivation-based consumer segments.

To address potential common method bias (CMB), both ex ante procedural remedies and ex post statistical assessment were employed, following the recommendations of Podsakoff et al. [67]. Procedural remedies implemented during survey design included the following: (1) ensuring respondent anonymity to reduce evaluation apprehension; (2) randomising question blocks to separate predictor and outcome measures; (3) varying scale formats across sections to reduce response patterning; and (4) adapting items from established literature to ensure clear and unambiguous wording. As an ex post assessment, Harman’s single-factor test was conducted by entering all multi-item scale variables into an unrotated principal components analysis. Additionally, discriminant validity was further assessed using the heterotrait–monotrait (HTMT) ratio for all pairs of multi-item constructs [68]. As an additional post-hoc diagnostic, a common latent factor/unmeasured latent method construct (CLF/ULMC) approach was estimated. However, the method factor proved to be empirically indistinguishable from the substantive variance, thus resulting in considerable loading instability. Consequently, the CLF/ULMC estimates were considered non-informative for the purposes of inference. As another consequence, the correlated uniqueness (CU) approach was retained as the primary sensitivity analysis for common method bias. The confirmatory correlated uniqueness (CU) evaluated whether any residual covariance attributable to shared measurement context could materially distort the substantive measurement structure. A baseline four-factor CFA model was specified for the four focal constructs (ATT_CM, INT_CM, TRNC, and GACM) using the 20 manifest indicators (Q10–Q21). A parsimonious CU-adjacent model was then estimated by freely allowing residual covariances only between adjacent items within each battery, reflecting plausible local dependencies induced by item proximity, repeated response formats, or shared wording context. Specifically, residual covariances were introduced within ATT_CM (six adjacent pairs), INT_CM (four adjacent pairs), TRNC (four adjacent pairs), and GACM (two adjacent pairs), yielding sixteen additional within-battery residual parameters in total. Model adequacy was evaluated using χ^2^, CFI, TLI, RMSEA, and SRMR, and the CU model was compared with the baseline CFA through a nested χ^2^ difference test. To ensure that the CU specification did not alter the substantive interpretation of the constructs, the stability of standardized factor loadings between the baseline and CU models was assessed using the mean and maximum absolute change in loadings across the 20 indicators, along with checks for sign reversals.

Patterns of missing data were assessed for all questionnaire items prior to analysis. Item-level missingness was low and did not exceed conventional thresholds that would justify the use of imputation-based procedures. Listwise, deletion was applied on a model-specific basis, meaning that respondents were excluded only from the particular analysis for which relevant item responses were missing, while remaining available for all other analyses. This approach ensured maximal retention of cases in each analytical step while maintaining internal consistency of the psychometric and regression models.

In order to assess dimensionality and construct validity, a series of exploratory and confirmatory factor analyses were performed. The adequacy of the samples was confirmed by an excellent global Kaiser–Meyer–Olkin value (KMO = 0.932), while Bartlett’s test of sphericity was highly significant (χ^2^ = 5543.91, *p* < 0.001), indicating that the correlation matrix was suitable for factor extraction. Exploratory factor analyses (EFAs) were conducted separately for each construct using principal-axis factoring with a one-factor solution and Promax rotation. The four scales exhibited unidimensionality with factor loadings exceeding 0.40 and acceptable uniqueness values.

Subsequent confirmatory factor analyses (CFAs) were estimated using maximum-likelihood estimation in semopy, with one-factor models specified for ATT, INT, TRNC, and GACM. The model’s fit was evaluated using three different indices: the comparative fit index (CFI), the Tucker–Lewis index (TLI), and the root-mean-square error of approximation (RMSEA). As anticipated for a three-item scale, the GACM model was identified (df = 0), resulting in global fit indices that were deemed to be non-informative.

Consequently, its adequacy was evaluated based on factor loadings and theoretical coherence.

### 2.3. Awareness Assessment and Predictive Modelling

The level of awareness surrounding CM was gauged by querying respondents on their prior familiarity with the concept. In light of the response options comprising “yes,” “no” and “I am not sure,” this was indicated as a binary. Responses of “yes” were assigned a value of 1 (aware), whereas “no” and “I am not sure” were assigned a value of 0 (not aware). In order to examine the sociodemographic predictors of awareness, a binary logistic regression model was estimated with gender, age, education, residence, employment, parenthood and household income serving as categorical predictors. The estimation of parameters was conducted by maximum likelihood using the Logit function in the statsmodels library. To evaluate multicollinearity, variance inflation factors (VIFs) were computed for all predictors; values below 5 were used as the threshold for acceptable collinearity. Model fit was assessed using the likelihood-ratio χ^2^ test, and McFadden’s pseudo-R^2^. Model calibration was additionally evaluated using the Hosmer–Lemeshow goodness-of-fit test (10-group specification). The calculation of odds ratios (ORs) and 95% confidence intervals (CIs) were undertaken to facilitate interpretation. Sensitivity analyses were conducted by confirming that all results were robust to the exclusion of cases with missing demographic information.

### 2.4. Cluster Analysis and Segment Derivation

Psychographic segmentation was undertaken to identify coherent subgroups of meat-eating consumers differing in their attitudinal, intentional and value-based profiles toward cultured meat. The basis for clustering was four standardised composite variables (ATT_CM, INT_CM, TRNC and GACM). In addition to this psychographic segmentation, a separate clustering procedure was conducted for conventional meat-purchase motivations using the eight Q7 items to derive behavioural segments reflecting how consumers prioritise sensory, ethical, health-related and economic attributes when buying conventional meat. Prior to the selection of the number of clusters, an exploratory evaluation of alternative solutions was conducted. For both the items pertaining to the motivation for purchasing meat (Q7.1–Q7.8) and the psychographic variables associated with cultured meat, candidate solutions ranging from k = 2 to k = 6 were estimated and compared using the silhouette coefficient, the Calinski–Harabasz index and the Davies–Bouldin index. Despite the fact that the two-cluster solutions yielded the highest silhouette values numerically, they lacked interpretative resolution and produced overly coarse partitions that were not aligned with theoretical expectations.

In terms of the motivations underpinning meat purchases, the three-cluster solution was found to offer the most coherent behavioural structure, demonstrating acceptable validity indices and clear differentiation between consumer groups. In the context of cultured-meat psychographics, the four-cluster solution exhibited a balanced statistical adequacy with conceptual differentiation, thus circumventing the instability and disproportionate cluster sizes observed in certain three- and five-cluster solutions. This four-cluster solution was deemed to offer the most meaningful structure for subsequent analyses and was therefore retained for all further work. Following the selection of k, the K-means clustering process was initiated, employing fifty random initialisations and a fixed random seed to ensure replicability. Prior to the implementation of the clustering process, all composite variables underwent a standardisation process that involved the transformation of their values to z-scores. Additionally, a listwise deletion approach was employed to address the presence of missing values. The quality of the clusters was evaluated using the silhouette coefficient, the Calinski–Harabasz index and the Davies–Bouldin index. In order to facilitate interpretation, cluster centroids in original scale units were also computed.

The stability of both clustering solutions was examined using two complementary approaches. The first approach relied on a bootstrap-like subsampling strategy. One hundred random subsamples, each comprising seventy per cent of the analytic dataset, were drawn independently. For each subsample, K-means clustering was re-estimated using the predetermined number of clusters (k = 3 for meat-purchase motivations and k = 4 for cultured-meat psychographics), and the resulting partitions were compared with the reference solution obtained from the full dataset. The adjusted Rand index (ARI) was utilised to quantify the correspondence between partitions. This procedure enabled an evaluation of the robustness of the discovered structures to sampling variability and furnished a distribution of ARI values for each domain. It was hypothesised that k = 2 could be a statistically efficient solution, as indicated by exploratory validity indices. Therefore, emphasis was placed on rigorously assessing the stability of higher-order segment structures. This was done to ensure that the chosen multi-cluster solutions were not artefactual or sensitive to sampling variability. The second approach evaluated stability through classification-based validation. Linear discriminant analysis (LDA) was utilised to predict cluster membership directly from the original variables forming the basis of each segmentation. The ten-fold cross-validation technique was employed to obtain an unbiased estimate of the predictive accuracy and to derive confusion matrices that summarised the separability of the clusters.

Although no formal a priori power analysis was conducted, the final sample size (*n* = 425) can be considered adequate for the analytical techniques applied. Specifically, the sample exceeds commonly recommended minimums for cluster analysis (at least 10 observations per clustering variable, [69]) and for logistic regression models (at least 10 events per predictor variable, [70]). These criteria suggest that the analyses were sufficiently supported by the available data.

## 3. Results

### 3.1. Sample Characteristics

The final analytic sample consisted of 425 adult Polish consumers who reported consuming conventional meat. This subsample was derived from an initial dataset comprising 483 completed questionnaires, with individuals who declared never or only very rarely consuming meat being excluded from the analysis. This restriction was applied to ensure conceptual alignment between the target population of cultured meat—positioned as a substitute for conventional meat—and the study sample. This allowed a more valid assessment of behavioural intentions and potential substitution patterns among current meat consumers. The sociodemographic profile of the resulting meat-eating sample is summarised in Table 1.

The sample population was predominantly female, with approximately two-thirds of the subjects being women (66.1%), while the remaining third were male (33.9%). The age distribution of the sample was relatively balanced between younger (18–34 years; 41.9%) and middle-aged adults (35–54 years; 41.1%), while older respondents (>54 years) accounted for 17.0% of the sample. Educational attainment was found to be strongly skewed towards higher education, with nearly three quarters of respondents (73.7%) reporting a high level of education, and only 10.1% and 16.2% in the low and medium categories, respectively. The majority of participants resided in urban areas, with 32.7% living in cities with a population exceeding 500,000 inhabitants and a further 38.0% in smaller cities, while 29.4% lived in rural areas. Household income was most frequently reported in the medium (41.0%) and low (36.7%) categories, with 22.3% of households reporting a high income. The survey revealed that just over half of respondents (54.1%) had children, and 68.0% were in employment. Notably, the “not working” category included full-time students and homemakers, rather than solely unemployed jobseekers. This accounts for the discrepancy between the reported non-working share (32%) and national unemployment statistics. The survey employed a non-probabilistic convenience sampling method using online distribution channels, including university mailing lists and social media platforms. As such, the sample was skewed toward younger, urban, and university-educated participants. This pattern reflects the demographic profile of individuals more likely to engage in digital surveys and to express interest in novel or prosocial topics such as emerging food technologies. While the data provide valuable insight into likely highly receptive consumers, the sample does not fully represent the general Polish meat-eating population. The sample displays the typical characteristics of online samples, exhibiting an over-representation of younger, urban, and highly educated consumers relative to the general Polish population.

### 3.2. Awareness of Cultured Meat and Its Sociodemographic Predictors

A binary logistic regression model was estimated to identify socio-demographic determinants of prior awareness (Q1) of cultured meat (0 = “no” or “I’m not sure,” 1 = “yes”). The model showed a statistically significant overall fit (likelihood-ratio χ^2^(12) = 85.82, *p* < 0.001) and a McFadden pseudo-R^2^ of 0.15, indicating that the predictors accounted for a non-trivial share of variance in awareness. Model calibration was adequate, as suggested by the Hosmer–Lemeshow goodness-of-fit test (χ^2^(8) = 8.28, *p* = 0.41).

Relative to the youngest age group (18–34 years), respondents aged 55 years and above were substantially less likely to have heard about cultured meat (OR = 0.19, 95% CI: 0.09–0.42, *p* < 0.001) (Table S4). In contrast, secondary (OR = 3.43, 95% CI: 1.08–10.87, *p* = 0.036) and especially university education (OR = 11.90, 95% CI: 2.37–37.38, *p* < 0.001) were positively associated with awareness when compared with primary education. Living in a city with more than 500,000 inhabitants increased the odds of prior awareness (OR = 2.91, 95% CI: 1.02–4.32, *p* = 0.045), whereas being in full-time employment was associated with lower odds compared with the reference category (OR = 0.48, 95% CI: 0.25–0.90, *p* = 0.022). Sex, the intermediate age group (35–54 years), residence in smaller cities or villages, parental status and income were not statistically significant predictors (all *p* > 0.05). Variance inflation factors did not indicate problematic multicollinearity, supporting the stability of the estimated coefficients.

The findings of this study indicate that awareness of cultured meat is concentrated among younger consumers, those with higher levels of education and those residing in urban areas. The complete model estimates can be found in Table S3 (Supplementary Materials).

### 3.3. Scale Reliability and Descriptive Statistics

The internal consistency statistics for all multi-item constructs utilised in the study are presented in Table 2. The psychometric performance of the scales was satisfactory and aligned with expectations for attitudinal and behavioural measures in food-technology research.

Attitudes toward cultured meat were measured with seven items capturing overall evaluation, perceived appeal and perceived appropriateness of cultured meat as a food product. Behavioural intentions were operationalised as the mean of five items referring to willingness to try, purchase and repeatedly buy cultured meat under realistic market conditions. Technological risk and naturalness concern (TRNC) was captured with five items referring to perceived unnaturalness, worry about technological complexity and discomfort with advanced food technologies; these items were reverse-coded to form a technological risk and naturalness concern (TRNC) index, in which higher scores indicate stronger concern. General acceptance of cultured meat was defined as the mean of three items addressing perceived need for cultured meat, its perceived superiority relative to other meat alternatives and its alignment with personal standards and values.

The seven-item attitude scale (ATT_CM) demonstrated acceptable reliability, with Cronbach’s α = 0.656 and a substantially higher McDonald’s ω = 0.821. While the alpha coefficient for ATT is slightly below the traditional 0.70 threshold, it is considered acceptable within the context of exploratory research addressing novel and complex psychological constructs [71]. The discrepancy between the two coefficients indicates a congeneric measurement model, where factor loadings vary across items; in such instances, ω serves as a more robust and accurate estimator of internal consistency than α [72], indicating that, despite modest heterogeneity among items, the underlying latent construct was measured with adequate coherence under a congeneric model. The mean score suggests that respondents expressed moderately positive evaluations of cultured meat, consistent with the positioning of cultured meat as an emerging but conceptually familiar innovation.

Behavioural intentions towards cultured meat (INT_CM) demonstrated excellent internal consistency, with α = 0.917 and ω = 0.938—values that are significantly above established benchmarks. This high reliability is indicative of the conceptual tightness of the willingness-to-try, purchase and recommend items. Descriptively, intentions were moderately positive, aligning with the broader pattern of cautious openness observed in the attitudinal indicators.

The five-item technological risk and naturalness concern (TRNC) scale demonstrated robust performance, with alpha = 0.821 and omega = 0.878, signifying substantial internal consistency despite the construct encompassing numerous facets of perceived artificiality, technological apprehension, and naturalness-related concerns. While the mean TRNC score was moderate overall, the scale demonstrated a relatively broad dispersion of responses (SD = 0.91). The item means ranged from 2.83 to 3.53, suggesting that respondents varied significantly in their perception of CM as technologically complex or insufficiently natural.

As anticipated for a concise instrument comprising three items, the general acceptance scale (GACM) yielded slightly diminished yet resilient reliability indices (α = 0.771, ω = 0.868), substantiating the adequacy of the items in encapsulating a shared evaluative dimension concerning the perceived societal and personal value of cultured meat. The mean score (M = 3.72, SD = 0.87) places respondents above the neutral midpoint, once more indicating a pattern of measured but genuine openness to the technology.

Discriminant validity was assessed using the heterotrait–monotrait (HTMT) ratio, which has been established as a superior criterion for evaluating the distinctness of latent constructs in variance-based structural equation modelling [70]. All HTMT values remained below the conservative 0.85 threshold, providing robust evidence for the empirical distinctness of the constructs. The highest HTMT value was observed between attitude toward cultured meat (ATT_CM) and general acceptance of cultured meat (GACM; HTMT = 0.830), followed by ATT_CM and intention (INT_CM; HTMT = 0.756), and ATT_CM and perceived technological risk and concerns (TRNC; HTMT = 0.717). The remaining construct pairs demonstrated lower HTMT values: INT_CM–GACM (0.740), TRNC–GACM (0.623), and INT_CM–TRNC (0.521). These results confirm that the constructs, while conceptually related within the domain of cultured meat acceptance, represent empirically distinct dimensions.

To address potential common method bias (CMB), both ex ante procedural remedies and an ex post statistical test were employed, following the methodological recommendations of Podsakoff et al. [69]. As an ex post statistical assessment, Harman’s single-factor test was conducted by entering all items from the multi-item scales (ATT_CM, INT_CM, TRNC, and GACM) into an unrotated principal components analysis. The first principal component accounted for 46.11% of the total variance, which is below the 50% threshold that would indicate substantial common method variance. In light of the Harman [69] single-factor test’s failure to indicate the dominance of a singular general factor (with the initial component accounting for 46.11% of the variance), it can be deduced that common method variance is unlikely to fully account for the observed covariance. Nevertheless, it is important to note that this test is regarded as a preliminary diagnostic tool only. To ensure a comprehensive assessment, the study incorporated a theory-driven sensitivity analysis, employing the correlated uniqueness (CU) approach as a methodological framework. The baseline four-factor CFA for the 20 indicators (Q10–Q21) yielded χ^2^ = 794.27 (df = 164), CFI = 0.8847, TLI = 0.8664, RMSEA = 0.0951, and SRMR = 0.0604. When local within-battery residual dependence was modelled via a parsimonious CU-adjacent specification (six residual covariances for ATT_CM, four for INT_CM, four for TRNC, and two for GACM; sixteen additional residual parameters in total), model fit improved markedly, with χ^2^ = 398.05 (df = 148), CFI = 0.9543, TLI = 0.9413, RMSEA = 0.0631, and SRMR = 0.0413 (AIC = 22,642.14; BIC = 22,893.37). The improvement over the baseline model was statistically significant (Δχ^2^ = 161.75, Δdf = 16, *p* < 0.001), and the CU model produced an admissible solution without negative residual variances.

Importantly, the CU specification did not substantively alter the measurement structure: standardized factor loadings remained highly stable relative to the baseline CFA, with a mean absolute change of |Δλ| = 0.034 and a maximum absolute change of |Δλ| = 0.110 across the 20 indicators, and no sign reversals were observed. Collectively, these results indicate that any additional shared variance is more plausibly attributable to local within-battery residual dependencies rather than to a dominant global method factor that would threaten the substantive interpretation of the constructs. Accordingly, the pattern of findings provides convergent evidence that common method bias is unlikely to account for the observed relationships in this dataset.

The multi-item constructs exhibited strong psychometric properties. The overall sampling adequacy was deemed to be excellent (KMO = 0.932), and Bartlett’s test of sphericity confirmed that the correlation structure was suitable for factor analysis (χ^2^ = 5543.91, *p* < 0.001). EFA was then employed to confirm the unidimensionality of each scale, with factor loadings ranging from −0.60 to −0.82 for ATT, −0.88 to −0.92 for INT, −0.65 to −0.77 for TRNC, and −0.41 to −0.86 for GACM.

The factorial validity of the constructs was further supported by CFA model fit indices. The initial model demonstrated an excellent fit (CFI = 0.985; TLI = 0.970; RMSEA = 0.110), followed by the TRNC model (CFI = 0.912; TLI = 0.822; RMSEA = 0.175) and the ATT model (CFI = 0.829; TLI = 0.743; RMSEA = 0.216). Although the RMSEA for the ATT scale was high (0.216), this index is known to be less reliable and often inflated in CFA models with a small number of items and few degrees of freedom. The construct’s validity is instead supported by robust factor loadings and the unidimensionality confirmed in EFA. As the GACM measurement model was found to be saturated (df = 0), it was not possible to compute confirmatory model fit indices. Consequently, construct validity was evaluated on the basis of internal consistency and factor loadings. The three items exhibited an excellent level of convergence, loading strongly on a single factor (|λ| > 0.57), and demonstrated a high degree of reliability. The absence of fit indices is acknowledged as a limitation, and future research is encouraged to expand the item pool to allow for a more differentiated modelling of evaluative components. Post-hoc modifications based on modification indices, such as the inclusion of error correlations, were deliberately omitted in the present analysis. This approach was adopted to maintain the theoretical parsimony of the unidimensional constructs and to prevent data-driven overfitting of the measurement models. Consequently, the models are reported in their original form to provide a transparent account of their performance in this specific sample. Overall, while I acknowledge the limitations in absolute fit for some scales, I conclude that the constructs exhibit adequate internal reliability and structural coherence suitable for subsequent segmentation.

Across all scales, item-total correlations were consistently within acceptable to strong ranges, providing further evidence of internal coherence. A limitation of the psychometric analysis is the high RMSEA for the attitude scale’s CFA model. While this may suggest a lack of perfect absolute fit, it is consistent with methodological findings on the behaviour of RMSEA in short scales [73], and the construct remains reliable for exploratory purposes. The discrepancy in fit indices between the scales, specifically the higher RMSEA for ATT (0.216) and TRNC (0.175) compared with INT (0.110), reflects the greater conceptual heterogeneity of attitudinal and risk-related constructs. While the intention items share close semantic proximity, the attitude items cover diverse evaluative facets (e.g., health, environment, and welfare), which introduces more residual variance in a strict CFA model despite the unidimensionality confirmed by EFA. Collectively, the reliability analyses confirm that the psychometric constructs used in the present study are conceptually coherent and suitable for subsequent segmentation.

### 3.4. Clustering and Segmentation

#### 3.4.1. Meat-Purchase Motivation Clusters

Segmentation based on conventional meat-purchase motivations used the eight Q7 items, which asked respondents how important specific aspects are when buying meat: Price, nutritional value, caloric content, taste, appearance, smell, environmental impact, and animal welfare. In order to ascertain the optimal number of clusters, a number of solutions (k = 2 to 6) were evaluated separately. Despite the two-cluster solution yielding a marginally elevated silhouette score (0.298 vs. 0.236), it resulted in excessively coarse groupings. The three-cluster solution offered a segmentation that was more interpretable, distinguishing between high-engagement consumers, sensory-driven consumers, and low-involvement individuals. The clustering validity indices indicated an acceptable structure (silhouette = 0.236; Calinski–Harabasz = 142.63; Davies–Bouldin = 1.47), supporting the retention of the three-segment model. The cluster sizes were found to be uneven but stable, with Cluster 0 comprising 52.1% of respondents, Cluster 1 36.5%, and Cluster 2 11.4%. This finding suggests the presence of one dominant and two more selective consumer groups.

The first and largest segment (*n* = 197) “high-engagement, quality-and-values driven consumers,” was characterised by generally high importance ratings across almost all aspects. Mean scores for taste, appearance and smell were close to the top of the scale, and nutritional value was also rated as very important (M = 4.85). In addition, these consumers attached relatively high importance to environmental impact (M = 3.97) and animal welfare (M = 4.24). Price and caloric content were evaluated as moderately to highly important but did not dominate the profile. This pattern suggests a group of multi-criteria, quality- and ethics-oriented meat buyers who simultaneously attend to sensory, health-related and ethical attributes when purchasing meat.

The second segment (*n* = 138) “sensory-focused, moderately engaged consumers,” showed a more selectively sensory profile. Taste, appearance and smell were again rated as very important (M = 4.48, M = 4.22 and M = 4.24 respectively); however, nutritional value, caloric content, environmental impact and animal welfare all received noticeably lower scores than in the first segment. Price was of moderate importance. This profile corresponds to a group of predominantly sensory-driven meat buyers, for whom the hedonic experience of eating meat is central, whereas health- and ethics-related aspects play a more secondary role.

The third and smallest segment (*n* = 43) “low-involvement, indifferent consumers,” exhibited uniformly low importance ratings across all eight aspects (range: 1.93–2.53). Neither sensory attributes, nor price, nor health- and ethics-related considerations stood out as particularly salient. This pattern is consistent with a low-involvement segment, in which meat purchase decisions are less strongly guided by specific evaluative criteria and may be more routine or opportunistic in nature.

**Figure 1:**
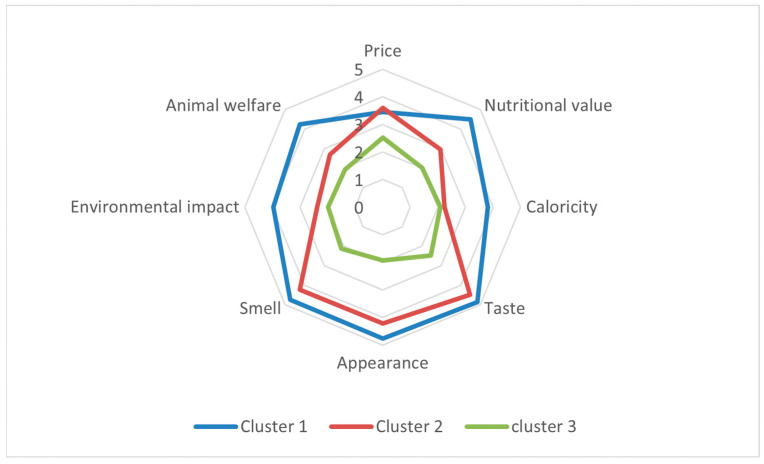
Comparative radar chart of attribute importance in meat-purchase decisions across consumer clusters.

As demonstrated in the radar chart (Figure 1), Cluster 1 forms a large, multidimensional polygon, Cluster 2 a narrower sensory-centred shape, and Cluster 3 a small, nearly circular profile, confirming clear visual distinctions among the segments.

A sociodemographic analysis revealed systematic differences between the three segments of consumers with different motivations for purchasing meat (Table 3). The association between gender and cluster membership was found to be statistically significant (χ^2^(2) = 49.60, *p* < 0.001, Cramér’s V = 0.36). The majority of female respondents (65.3%) were classified into the high-engagement, quality-and-values-driven cluster (Cluster 1), whereas only 27.8% of male respondents belonged to this segment. Conversely, a significantly higher proportion of males were observed in the sensory-focused, moderately engaged cluster (Cluster 2; 57.1% of males vs. 25.3% of females). The low-involvement, indifferent cluster (Cluster 3) attracted relatively small proportions of both genders, but again with a slight male predominance (15.0% vs. 9.4%).

Furthermore, an association was observed between age and cluster membership (χ^2^(4) = 39.76, *p* < 0.001, Cramér’s V = 0.23). The younger consumer demographic (18–34 years) was predominantly distributed between the two more engaged segments, with approximately 57% assigned to Cluster 1 and 38% to Cluster 2, and a mere 4.7% falling into the low-involvement segment. Among respondents within the 35–54 age group, Cluster 1 maintained its position as the predominant category (59.3%), while Cluster 3 exhibited a notable increase in its share (14.5%). In contrast, the 55+ age group exhibited a distinctly divergent pattern, with a mere 21.0% of respondents classified as high-engagement consumers. The remaining majority were predominantly allocated to either the sensory-focused Cluster 2 (56.5%) or the indifferent Cluster 3 (22.6%), suggesting that older consumers of meat were less inclined to attach significant importance to a comprehensive array of meat attributes.

The data demonstrated a similar gradient in educational attainment (χ^2^(4) = 43.05, *p* < 0.001, Cramér’s V = 0.24). Respondents who had received only primary education were found to be present in low numbers within the high-engagement cluster (7.1%), with the majority of this group being allocated to Cluster 2 (61.9%) or Cluster 3 (31.0%). Amongst those with secondary or university education, Cluster 1 became clearly dominant, encompassing 54.7% and 58.5% of these respondents, respectively, while the indifferent segment shrank to around 9–11%. Consequently, consumers who attach comparatively little importance to meat attributes tend to be less well-educated, whereas the most engaged segment is characterised by higher levels of formal education.

The findings indicate that neither place of residence nor employment status were found to be significantly associated with cluster membership (both *p* > 0.05). This suggests that urban–rural location and working versus non-working status do not materially differentiate meat-purchase motivation profiles in this sample. The study found a modest yet statistically significant correlation (χ^2^(2) = 8.61, *p* = 0.013, Cramér’s V = 0.15). The proportion of parents was higher in the low-involvement cluster (15.6% of parents vs. 7.0% of non-parents assigned to Cluster 3), whereas respondents who were childless were somewhat more likely to belong to the sensory-focused Cluster 2. The association between household income and cluster membership was only marginal (χ^2^(4) = 8.71, *p* = 0.069, Cramér’s V = 0.11). There was a tendency for lower-income respondents to be slightly over-represented in the indifferent segment and higher-income respondents in the high-engagement cluster.

The results of the profiling indicate that consumers who are highly engaged, quality-driven and values-driven are more often female, middle-aged or younger, and better educated. In contrast, sensory-focused consumers and especially those in the low-involvement segment are more prevalent among men, older respondents and those with lower educational attainment.

#### 3.4.2. Cultured Meat Psychographic Clusters

**Figure 2:**
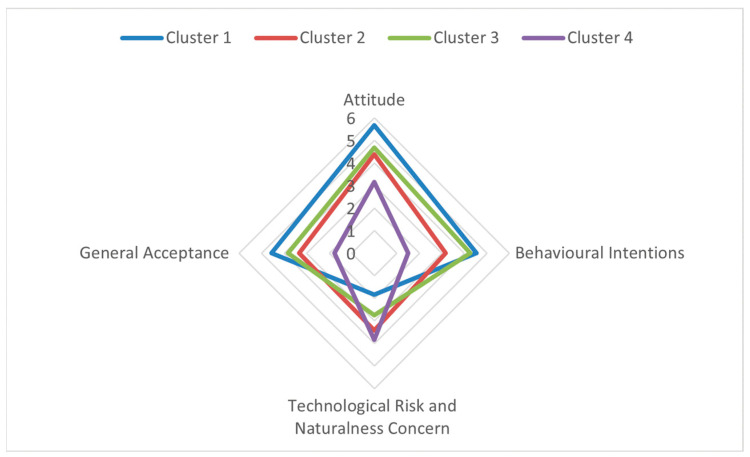
Psychographic profiles of the four consumer segments across attitudes, behavioural intentions, technological risk and naturalness concern (TRNC), and general acceptance of cultured meat.

In the context of the psychographic segmentation of cultured meat, the k-means clustering method was employed to analyse the four composite indices that encapsulated the following: attitudes (ATT_CM), behavioural intentions (INT_CM), technological risk and naturalness concern (operationalised here as neophobia) and general acceptance (GACM). To determine the optimal number of clusters, several solutions (k = 2 to 6) were evaluated. The four-cluster solution (k = 4) was determined to provide the optimal balance between statistical adequacy and conceptual differentiation, as indicated by the validity indices (silhouette coefficient = 0.25; Calinski–Harabasz index = 222.25; Davies–Bouldin index = 1.32). The distribution of cluster sizes exhibited a moderate imbalance, yet remained interpretable, with segments comprising 24.9%, 32.0%, 36.5%, and 6.6% of the respondents, respectively. The corresponding profiles are visualised in the radar chart in Figure 2, which highlights clear separation between segments along all four psychographic dimensions.

The first segment (*n* = 106) is “highly receptive consumers.” The group in question demonstrated a predominantly positive orientation towards cultured meat. Their radar profile is represented by the broadest polygon in Figure 2, reflecting very high attitudes (M = 5.67), behavioural intentions (M = 4.54) and general acceptance (M = 4.57), combined with the lowest TRNC score (M = 1.83). This segment demonstrates a notable degree of positive emotional, evaluative, and behavioural readiness to adopt cultured meat, accompanied by minimal technological apprehension.

The second segment (*n* = 136) comprised “concerned ambivalents.” Despite the moderately positive nature of the attitudinal evaluations (M = 4.38), the behavioural intentions exhibited a notable decline (M = 3.16), and the general acceptance was only moderate (M = 3.33). It is noteworthy that this cluster demonstrated elevated TRNC levels (M = 3.43), indicating that discomfort with technological novelty and perceived unnaturalness functions as a significant impediment to the translation of positive attitudes into behavioural readiness. This group therefore embodies a form of evaluative-behavioural inconsistency driven by risk-related concern.

The third and largest segment (*n* = 155) is “cautious optimists.” The respondents in this segment demonstrated elevated attitudes towards cultured meat (M = 4.69) and concomitantly robust behavioural intentions (M = 4.25). Furthermore, the level of general acceptance was found to be above the midpoint (M = 3.83). However, their TRNC scores (M = 2.75) were higher than those observed in Cluster 1, indicating a degree of residual technological reservation. The overall profile indicates a group that is favourable towards cultured meat, albeit with a degree of moderation due to a sense of caution.

Finally, Cluster 4 constituted a diminutive yet distinctly delineated collective of pronounced sceptics. This segment was characterised by the lowest attitudinal evaluations (M = 3.15), very low behavioural intentions (M = 1.51), and minimal general acceptance (M = 1.77), combined with the highest TRNC scores (M = 3.84). The respondents to this survey demonstrated a clear reluctance to embrace cultured meat, regardless of its potential benefits. This resistance was further compounded by their concerns regarding the technological aspects of the product. In conclusion, this segment can be regarded as exhibiting a clear rejection orientation.

The four-cluster solution displayed acceptable validity indices. Although the two- and three-cluster solutions yielded slightly higher silhouette values, they produced overly coarse groupings that merged conceptually distinct response patterns. The four-cluster solution maintained adequate separation between segments while providing a richer and more interpretable segmentation structure, particularly by distinguishing between enthusiastic, moderately positive, ambivalent and rejecting consumers. Collectively, these clusters illustrate a well-differentiated psychographic landscape, spanning from high-readiness highly receptive consumers through ambivalent and cautiously positive consumers to a minority of strong opponents. This distribution underscores the importance of considering both evaluative dispositions and underlying technological concerns when interpreting consumer responses to cultured meat.

The sociodemographic and awareness profiles of the four psychographic segments were examined using cross-tabulation and chi-square tests (Table 4).

Awareness-related variables demonstrated the strongest associations with segment membership. The respondents’ prior awareness of cultured meat (Q1) exhibited a significant correlation with their cluster allocation (χ^2^(6) = 52.81, *p* < 0.001, Cramér’s V = 0.25). Among those who had already heard of cultured meat, over one third were classified as highly receptive consumers (36.6%) and a similar proportion as cautious optimists (37.0%), whereas only 4.8% belonged to the strong sceptics segment. Conversely, among respondents who professed a lack of familiarity with cultured meat, concerned ambivalents constituted the predominant group (41.3%), while the proportion of pronounced sceptics more than doubled to 11.6%. A comparable pattern was observed for self-rated knowledge about cultured meat (Q2; χ^2^(12) = 64.27, *p* < 0.001, V = 0.22) and familiarity with the production process (Q3; χ^2^(6) = 44.66, *p* < 0.001, V = 0.23). Respondents who self-rated their knowledge as either well-informed or very well-informed were predominantly found among highly receptive consumers and cautious optimists. In contrast, those reporting no or poor knowledge were more frequently classified as concerned ambivalents or strong sceptics.

The present study examined the association between cluster membership and the willingness to replace conventional meat with meat substitutes. The latter were defined as plant-based products or edible insect alternatives. The results demonstrated a strong association between these variables (χ^2^(6) = 81.14, *p* < 0.001, Cramér’s V = 0.31). Among respondents who selected “No” on the basis of complete unwillingness to replace conventional meat with these substitutes, concerned ambivalents represented the largest share (41.2%), while strong sceptics constituted an additional one fifth (20.2%). Conversely, highly receptive consumers and cautious optimists were conspicuously under-represented in this category. For those who selected “Yes, but not much,” there was a shift in distribution towards the more open segments, with cautious optimists and highly receptive consumers playing a more prominent role. The pattern was most pronounced among respondents who answered in the affirmative, indicating a clear readiness to replace conventional meat with plant-based or insect-based substitutes. Within this group, 38.0% of respondents were classified as cautious optimists, while 38.0% were designated as highly receptive consumers. Strong sceptics accounted for fewer than 2% of cases. Taken together, these results confirm that the four segments differ not only in attitudinal orientation towards cultured meat but also in their broader behavioural readiness to substitute conventional meat with alternative proteins, spanning both plant-based and insect-based options.

A number of sociodemographic variables were found to be associated with membership of a psychographic segment. The study found no statistically significant associations between gender and place of residence with the clusters (Q22 and Q25, both *p* > 0.05). Parenthood exhibited a very weak, non-significant association (Q27; *p* = 0.28). In contrast, age (Q23), educational attainment (Q24), employment status (Q26) and household income (Q28) exhibited meaningful, albeit small-to-moderate, associations (all *p* < 0.05; Cramér’s V between 0.14 and 0.23). In the present study, respondents between the ages of 18 and 54 were found to be more likely to be classified as highly receptive consumers or cautious optimists, in contrast to the older age group (55+ years), which was found to be disproportionately represented among concerned ambivalents (63.4%) and, to a lesser extent, among cautious optimists. This suggests that older respondents were less likely to be classified as highly receptive consumers. Respondents with a university education were predominantly located in the positive segments (29.7% highly receptive consumers and 38.3% cautious optimists, compared with only 4.5% strong sceptics), while those with only primary schooling were most often classified as concerned ambivalents (67.4%) or strong sceptics.

The employment status exhibited a modest yet statistically significant association (χ^2^(3) = 8.54, *p* = 0.036, V = 0.14). Amongst non-working respondents, concerned ambivalents constituted the largest group (40.4%), whilst working respondents were more evenly distributed between the three more positive segments, with cautious optimists being the most frequent (36.7%). Furthermore, a correlation was identified between household income and cluster membership (χ^2^(6) = 25.08, *p* < 0.001, V = 0.17). Respondents from higher-income households (above 10,000 PLN) were most commonly classified as cautious optimists (41.1%) or highly receptive consumers (29.5%), whereas those from the lowest income category (up to 5000 PLN) were more often concerned ambivalents (44.9%) and less often highly receptive consumers (16.7%). These patterns should not be interpreted as formal moderation effects, but rather as descriptive differences in segment composition across sociodemographic groups, as no interaction terms were formally tested in the present analyses.

Due to the non-probabilistic online recruitment strategy, the sample is skewed toward younger, highly educated, and urban respondents. This demographic composition may influence both the prevalence of segments (e.g., over-representation of more innovation-exposed groups, such as highly receptive consumers and cautious optimists) and the stability and interpretability of smaller clusters (e.g., strong sceptics). Consequently, the identified segment structures should be interpreted as indicative of attitudinal configurations within receptive populations rather than as representative of the broader Polish meat-eating population.

When considered as a whole, these profiles indicate that highly receptive consumers and cautious optimists tend to be better informed about cultured meat, more willing to replace conventional meat, and somewhat younger, better educated and more affluent. Concerned ambivalents and strong sceptics, in turn, are characterised by lower awareness and knowledge, reduced willingness to substitute, and particularly in the case of sceptics, less favourable socioeconomic characteristics.

#### 3.4.3. Cluster Stability and Classification-Based Validation

The initial investigation into cluster stability was conducted utilising a bootstrap resampling approach, founded upon the adjusted Rand index (ARI). For the three meat-purchase motivation clusters, ARI values across the 100 bootstrap samples were consistently high, with a mean of 0.86 (median 0.87; range 0.53–1.00). In 84% of the re-samples, the ARI exceeded 0.80, and more than one third of the replications reached values above 0.90. This finding indicates that the three-segment structure for conventional meat motives was highly robust to sampling variation. The four psychographic segments derived from cultured meat exhibited a remarkably consistent pattern, with an average ARI of 0.87 (median 0.91; range 0.43–0.99). In this particular instance, 83% of the bootstrap solutions yielded ARI values greater than 0.80, with nearly 60% achieving values above 0.90. On occasion, lower ARI values were observed for both segmentation schemes. However, these represented isolated reallocations of a small number of boundary cases rather than systematic instabilities in the overall cluster configuration.

A secondary line of evidence was obtained from classification-based validation using linear discriminant analysis (LDA). In the context of the meat-purchase segmentation, 10-fold cross-validation yielded a mean predictive accuracy of 0.94, closely mirroring the overall resubstitution accuracy derived from the confusion matrix (93.7%). The accuracies for the three clusters were 97.9%, 89.9% and 86.0%, respectively. The majority of misclassifications occurred between the two more engaged segments, with only a few cases from the low-involvement cluster being assigned elsewhere. In the context of the cultured-meat psychographic segmentation, LDA attained an even higher mean cross-validated accuracy of 0.96, with cluster-wise accuracies of 94.3%, 94.9%, 97.4% and 92.9% for the four respective segments. Misclassifications were again rare and predominantly involved neighbouring clusters along the acceptance–concern continuum. When considered as a whole, the results of the bootstrap ARI and LDA demonstrate convergent evidence that both the behavioural (i.e., meat purchase) and psychographic (i.e., cultural meat consumption) segmentations are stable and internally coherent structures, rather than artefacts of a particular sample split.

#### 3.4.4. Overlap Between Meat-Purchase Motivation Clusters and Cultured-Meat Psychographic Segments

A cross-classification of the three meat-purchase motivation clusters with the four cultured-meat psychographic segments revealed a statistically significant but modest association between the two segmentation structures (χ^2^(6) = 22.39, *p* = 0.001; Cramér’s V = 0.17; Theil’s U = 0.025). Despite the non-random nature of the relationship, the finding that U was at a very low value suggests that respondents’ conventional meat-purchase profiles only explained a small fraction of the uncertainty surrounding their psychographic orientation towards cultured meat.

When examined from the perspective of the meat-purchase motivation clusters, the majority of respondents across all three groups were classified into the more positive cultured-meat segments. In the high-engagement, quality-and-values-driven cluster, 36.5% of respondents were classified as cautiously optimistic CMs, while 32.0% were identified as highly receptive consumers. A mere 6.6% of respondents belonged to the strong sceptics category. A similar pattern was observed among sensory-focused consumers, where ambivalent and cautiously optimistic respondents predominated (40.6% and 39.1%, respectively), while the rejectionist segment accounted for just 5.8%. In the low-involvement meat cluster, where overall engagement with meat attributes was weakest, the majority of respondents were distributed across the moderately positive CM segments, and less than 3% were classified as strong sceptics.

Conversely, an examination of the data from the opposite perspective reveals that each cultured-meat segment encompasses members from multiple meat-motivation clusters. This finding serves to substantiate the notion that the psychographic segmentation cannot be reduced to conventional meat-purchase motives. The highly receptive consumers who demonstrated a high level of enthusiasm were predominantly, although not exclusively, drawn from the two clusters that exhibited a greater degree of engagement, with these groups accounting for 36–38% of the sample. Concerned ambivalents and cautious optimists exhibited a balanced distribution across the three meat segments, whereas the strong sceptics—although numerically small—were most likely to originate from the high-engagement or sensory-focused meat clusters and only rarely from the low-involvement cluster.

**Figure 3:**
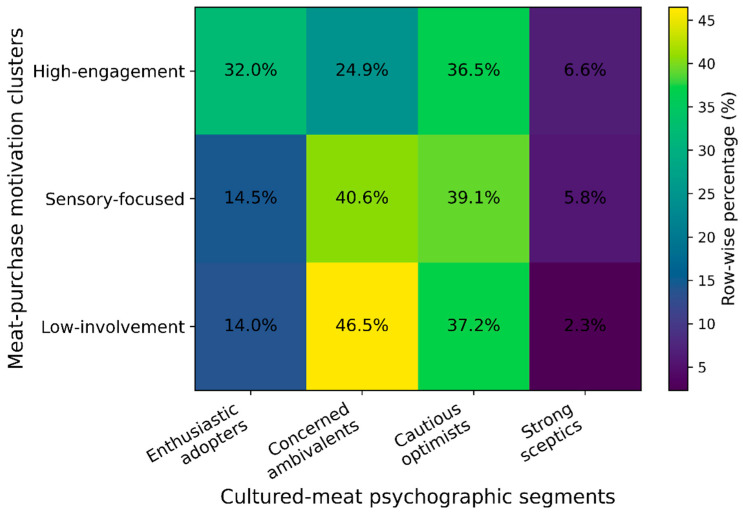
Heatmap of the overlap between meat-purchase motivation clusters and cultured-meat psychographic segments (row-wise percentages).

The overall pattern of the cross-classification is visualised in Figure 3, which presents the row-wise percentage distribution of cultured-meat segments within each meat-purchase motivation cluster. As demonstrated in Figure 3, the heatmap indicates that all three clusters of meat-purchase motivation are predominantly associated with the more positive segments of cultured meat. The high-engagement cluster demonstrates the strongest alignment with the highly receptive and cautiously optimistic segments, whereas the sensory-focused and low-involvement clusters display a similar pattern, albeit with slightly higher proportions of ambivalent consumers. Across all clusters, the proportion of strong sceptics remains negligible, thereby reinforcing the finding of limited correspondence between conventional meat-purchase motives and psychographic responses to cultured meat.

Taken together, these results demonstrate partial but limited structural correspondence between behavioural meat-purchase motives and psychographic evaluations of cultured meat. Consequently, the propensity for cultural meat is not restricted to consumers who place a lower value on conventional meat attributes; rather, it is pervasive among all categories of meat buyers, particularly those who are already profoundly engaged with meat quality, sensory attributes and ethical considerations.

## 4. Discussion

The findings of this study provide a comprehensive and empirically grounded picture of how Polish meat-eating consumers perceive, evaluate and position cultured meat within their broader food-choice frameworks. The results of the study indicate a pattern of cautious yet authentic openness. Attitudes towards cultured meat were moderately positive, behavioural intentions were above the scale midpoint, and general acceptance indicated a recognition of the potential societal value of this technology. These findings are consistent with the emerging consensus in recent international research, where consumers tend to express evaluative openness paired with behavioural reservation [26,27,28]. Comparable levels of cautious openness have also been reported in the United States [74], the United Kingdom [13], Belgium [75] and Serbia [76] although cross-country variation remains substantial. The present study contributes to the extant literature on this subject in two ways. Firstly, it provides evidence from a Central European context that is still underrepresented in global cultured-meat scholarship. Secondly, it situates these attitudes within consumers’ habitual motivations for purchasing conventional meat.

The results on awareness underscore the significant role of informational and knowledge-related variables in cultured-meat acceptance. Respondents who had previously encountered the concept of cultured meat, or who rated themselves as knowledgeable, were substantially more likely to belong to the positive segments—highly receptive consumers and cautious optimists. This finding is consistent with global literature emphasising familiarity as a critical determinant of openness to cellular agriculture technologies [77,78]. Analogous trends have been recorded in China and Belgium [26] and Italy [27], where prior exposure significantly increases the willingness to try cultured meat. The present Polish data thus serve to reinforce the argument that early communication strategies, which include explanations of production methods, safety measures, and potential benefits, may effectively shift consumers away from an uninformed state of scepticism.

The findings further demonstrate that the technological risk and naturalness concern (TRNC) persists as a pivotal impediment to behavioural engagement. These results are further supported by recent evidence from a multinational [79], which compared consumer acceptance of multiple emerging food technologies across several cultural contexts. In that study, cultured meat consistently ranked among the least accepted innovations, primarily due to perceived unnaturalness, technological unfamiliarity, and concerns related to safety and sensory unpredictability. The authors also emphasised that consumers struggled to imagine how such a product could be incorporated into everyday eating practices. This is an interpretative barrier closely aligned with the elevated TRNC scores observed among the concerned ambivalents in our sample. Moreover, the study reported significant cross-cultural variation, with acceptance levels higher in technologically progressive or food-security-oriented regions such as India and Singapore, and lower in countries with strong traditional meat cultures, such as Australia and the United States. This pattern places the Polish consumer landscape within a broader global trajectory, in which cautious interest coexists with deep-rooted reservations about naturalness and technological intervention. Concerned ambivalents constituted over one-third of the sample and exhibited a profile characterised by moderately positive attitudes but comparatively low intentions, driven in part by elevated TRNC scores. This evaluative–behavioural disconnect mirrors the tension observed in recent studies from Romania [29] and Croatia [80], where cultured meat elicits ethical or environmental approval yet triggers discomfort related to technological novelty and the perceived loss of naturalness. Broader socio-cognitive interpretations of cultured meat, as documented by Fasanelli (2025) [81]—who argues that CM evokes hybrid representations oscillating between “food” and “technology”—help to explain why consumers may support the idea of cultured meat but still hesitate to engage with it at the level of purchase or consumption.

The segmentation analysis provides further insight into this interpretative landscape. A recent systematic review [82] offers a valuable global perspective that aligns closely with the present findings. The review demonstrates that, in developed countries, acceptance of cultured meat is primarily shaped by familiarity, perceived naturalness, and food technology neophobia, whereas in developing regions environmental and food-security motivations are more prominent drivers. This cross-national evidence helps contextualise the Polish consumer landscape observed in this study. Despite moderately positive attitudes and conditional openness among Polish consumers, technological unfamiliarity and concerns about naturalness emerged as central inhibiting factors, mirroring the dominant barriers identified in high-income countries. The convergence between our psychographic segmentation and the global patterns described in the review suggests that Poland follows a typical acceptance trajectory for technologically advanced protein innovations within developed, meat-centric food cultures. The analysis identified four distinct psychographic segments: highly receptive consumers, cautious optimists, concerned ambivalents and strong sceptics. Each segment is characterised by unique configurations of attitudes, intentions and technological concerns. These segments bear a strong resemblance to those identified in multinational studies [28,30,31], where pro-innovation consumers consistently contrast with highly sceptical minority groups. It is noteworthy that the proportion of strong sceptics in the present study was minimal (6.6%), a finding that aligns with the South American and Middle Eastern trends documented by Mendes [30] and Khaleel [31], respectively. This figure is marginally lower than the observations recorded in Italy or Western Europe [27]. This may suggest that Polish meat consumers, particularly younger and better-educated adults, are relatively open to alternative proteins when presented with a credible technological narrative.

A pivotal finding of this study is the integration of psychographic segmentation with behavioural segmentation, derived from motivations for purchasing conventional meat. The overlap between the three behavioural meat-purchase clusters and the four cultured-meat psychographic segments was statistically significant but modest, as evidenced by both Cramér’s V and Theil’s U. This finding suggests that, although individuals who prioritise ethical and environmental attributes when purchasing conventional meat are somewhat more likely to be open to cultured meat, the explanatory power of meat-purchase motivations alone is limited. It is important to note that, due to the cross-sectional nature of the data, it is not possible to draw causal inferences. While the observed associations provide insight into possible relationships between conventional meat-purchase motives, attitudes, and behavioural intentions, the directionality of these associations remains uncertain. It is also plausible that positive attitudes toward cultured meat may influence retrospective evaluations of one’s ethical motives for purchasing meat, rather than the reverse. Consequently, all interpretations should be understood within the limits of a correlational framework. Although the present study identified statistically significant associations between motivations, attitudes, and behavioural intentions, the cross-sectional nature of the data precludes testing competing causal models. Future research should apply structural equation modelling (SEM), mediation analyses, or longitudinal and cross-lagged panel designs to examine the directionality and potential feedback mechanisms in these relationships.

These results call into question the assumptions present in parts of the alternative-protein literature, which often imply that ethical or sustainability-driven omnivores may naturally transition towards cultured meat [83,84,85]. The findings indicate that acceptance of cultured meat cannot be inferred solely from existing meat-choice heuristics. Instead, consumers’ responses to cellular agriculture products involve additional psychological dimensions, such as perceptions of technological naturalness, familiarity, trust and affective comfort. These dimensions are not captured by conventional meat-purchase motives.

The integration of cultured meat with other alternative proteins offers further nuance. It was found that respondents who declared a willingness to replace conventional meat with plant-based or insect-based substitutes were disproportionately represented among the two most positive segments of the cultured-meat population. This outcome aligns with international findings that openness to novel proteins tends to generalise across product categories and reflects broader dietary flexibility [11,86,87]. Nevertheless, the present study also demonstrates that a willingness to engage with plant-based or insect-based alternatives is not fully related to openness to cultured meat, indicating that the latter triggers unique concerns and considerations, particularly related to technological unfamiliarity. This underscores the necessity for bespoke communication and consumer education strategies, as opposed to presuming cross-category spillover effects.

The findings of this study demonstrate that sociodemographic factors have a significant impact on the outcomes under investigation. The findings of this study demonstrate that younger consumers, those with higher educational attainment and higher income levels, were more likely to belong to positive segments. This pattern is consistent with the results of studies conducted in China [88], Greece [28], Italy, and Brazil [30]. These demographic gradients are probably indicative of differential exposure to technological innovation, varying levels of trust in scientific institutions and diverse motivations related to sustainability and ethical consumption. The findings emphasise the necessity of formulating communication strategies that are tailored to older and less technologically confident consumers. These consumers may necessitate more explicit assurances regarding the naturalness, safety and societal benefits of the technology.

The findings of this study have several practical implications for stakeholders aiming to introduce cultured meat into the Polish market. The most promising early-stage consumer base is constituted by highly receptive consumers and cautious optimists, who together represent approximately 60% of the sample. It is recommended that communication strategies, which target these groups, place emphasis on the environmental benefits of cultured meat, improvements in animal welfare, customisable nutrition, and the role of cultured meat in a broader protein transition portfolio. In the case of individuals who harbour ambivalent feelings, transparent communication regarding technological processes, safety assessments and regulatory oversight may help to allay concerns. It is important to note that strong sceptics are unlikely to be receptive at the early stages of development. Therefore, they should not be considered a primary target market until a greater degree of societal legitimisation has been achieved. These recommendations are in alignment with international insights regarding targeted communication, influencer credibility and emotional framing [58,89,90].

Several limitations of the present study should be acknowledged when interpreting the findings. First, the sample was recruited through online channels, including university mailing lists and social media platforms, which resulted in a non-probabilistic convenience sample with notable demographic biases. Specifically, the sample was skewed toward younger, university-educated (73.4%), and urban-dwelling respondents, with an over-representation of female participants (66.1% female, 33.9% male). Although this demographic profile is common in online survey research on novel food technologies [62,66], it limits the generalisability of the findings to less educated, older, rural, or male-dominant segments of the Polish meat-eating population. Post-hoc statistical weighting was not applied, as such adjustments to non-probability samples can introduce additional bias rather than reduce it, particularly when the selection mechanism is correlated with the outcome variables of interest [91]. Consequently, the findings should be interpreted as characterising likely highly receptive segments, individuals more likely to engage with digital surveys and to express interest in novel or prosocial topics—rather than the general Polish meat-eating population. Future research should employ probability-based or quota sampling procedures, potentially with stratified sampling by gender, education, and geographic location, to enhance population representativeness and enable more robust demographic comparisons.

Second, the cross-sectional design precludes causal inference. Although the analytical framework implies a directional relationship from conventional meat-purchase motivations to cultured meat acceptance, the possibility of reverse causality cannot be excluded. For instance, it is plausible that respondents with pre-existing positive attitudes toward cultured meat may retrospectively reframe or re-evaluate their ethical motivations for purchasing conventional meat. Definitive causal ordering cannot be established without longitudinal data or experimental designs. Future research should apply structural equation modelling (SEM) with competing causal pathways, mediation analyses, or cross-lagged panel designs to examine the directionality of these relationships more rigorously.

Third, the study relied on a single-source, self-report survey, so method-related variance cannot be entirely excluded. A parsimonious correlated uniqueness sensitivity analysis, modelling local within-battery residual dependence between adjacent items, improved model fit while leaving standardised factor loadings essentially unchanged (mean |Δλ| = 0.034; max |Δλ| = 0.110; no sign reversals), suggesting that any additional shared variance is more plausibly local rather than attributable to a dominant global method factor. Nonetheless, future research should incorporate stronger design-based controls for common method bias, such as including a theoretically unrelated marker variable and/or introducing temporal separation between predictor and outcome measures [69].

Fourth, from a psychometric perspective, the attitude toward cultured meat scale (ATT_CM) demonstrated a Cronbach’s alpha coefficient (α = 0.656) slightly below the conventional 0.70 threshold, although McDonald’s omega (ω = 0.82) indicated adequate composite reliability. Furthermore, the CFA model for this scale exhibited an elevated RMSEA value (0.216), which, while acknowledged to be biased toward over-rejecting models with a small number of items and low degrees of freedom [75], suggests that the constructs should be treated as exploratory tools requiring further refinement rather than fully validated instruments. Future research should consider applying more sophisticated measurement approaches, such as exploratory structural equation modelling (ESEM), which integrates the flexibility of exploratory factor analysis with the hypothesis-testing capabilities of confirmatory approaches [92]. ESEM may be particularly useful for refining scales that exhibit modest cross-loadings or multidimensional tendencies.

Fifth, a methodological consideration concerns the informational note provided to participants prior to attitude measurement. As documented in a recent scoping review [66], the majority of studies on cultured meat acceptance provide definitional information to participants due to low public awareness. However, the information provided in the present study emphasised potential benefits (environmental sustainability, animal welfare) without presenting potential concerns (energy consumption, use of foetal bovine serum, regulatory uncertainty). Following Slovic’s [93] theory of preference construction, this positively framed information may have influenced responses in a favourable direction, particularly among the approximately 30% of respondents who reported no prior awareness of cultured meat. The attitudes reported should therefore be interpreted as reactions to a specific, positively framed value proposition rather than to cultured meat as a technology in general. Future research should employ balanced or comparative framing conditions to disentangle genuine acceptance from information-induced priming effects.

Sixth, the study measured behavioural intentions rather than actual purchase or consumption behaviour. The well-documented intention–behaviour gap in consumer research [94,95] suggests that stated willingness to try or purchase cultured meat may not translate into actual behaviour when the product becomes commercially available. The gap between stated intentions and actual behaviour is particularly pronounced for ethical consumption decisions, where contextual factors such as price, availability, social norms, and habitual patterns may override stated preferences. Future research should incorporate field experiments, revealed preference methods, or longitudinal tracking of actual purchase behaviour to validate the predictive utility of the identified consumer segments.

Finally, while the study provides valuable insights into the Polish consumer context, findings may not generalise to other cultural or regulatory environments with different food traditions, levels of meat attachment, or institutional frameworks governing novel food technologies. Cross-cultural replication studies would strengthen the external validity of the proposed motivational framework and enable more nuanced understanding of context-specific barriers and facilitators of cultured meat acceptance. It is recommended that future research incorporate experimental and longitudinal designs, sensory testing and price-sensitivity assessments, as well as comparative studies across Central and Eastern Europe. In view of the rapid expansion of the extant literature on cultured meat, particularly in 2025, future research should also incorporate emerging insights on regulatory evolution, LCA outcomes, nutrient-profile engineering and industrial-scale production.

Given the limited explanatory power of conventional meat-purchase motivations observed in this study, future research should incorporate additional psychosocial predictors that have demonstrated relevance in food technology acceptance contexts. These include trust in food governance and scientific institutions, disgust sensitivity, moralised eating orientations (e.g., ethical identity, moral conviction about animal agriculture), and broader food technology attitudes such as general neophobia and perceived benefit–risk trade-offs. Furthermore, future studies should explicitly compare acceptance patterns across multiple alternative protein categories, including plant-based proteins, hybrid meat products, and precision fermentation-derived ingredients to assess whether cultured meat acceptance reflects a broader ‘alternative protein openness’ portfolio or constitutes a distinct technology-specific response.

When considered as a whole, the findings position Poland within the broader global landscape of emerging cultured-meat markets. It is suggested that, while behavioural anchors rooted in conventional meat purchasing provide partial explanatory value, acceptance of cultured meat is best understood as a multidimensional psychological construct shaped by technological perceptions, familiarity, ethical considerations and socio-demographic context. The integration of behavioural and psychographic segmentation, alongside a foundation in the most recent international developments, renders this study a valuable addition to the field. It provides a nuanced and timely contribution to the understanding of the potential trajectory of cultured meat in a nation with a robust meat-eating tradition and a growing interest in sustainable food technologies.

## 5. Conclusions

It is imperative to interpret the ensuing conclusions within the confines of a cross-sectional, correlational design. The observed associations between conventional meat-purchase motivations and cultured meat acceptance cannot be interpreted as causal or predictive relationships, and the results are specific to the Polish cultural and regulatory context, limiting generalisability to other national settings.

This study provides an integrated examination of how Polish meat consumers perceive cultured meat and to what extent these perceptions relate to their established motivations for purchasing conventional meat. The findings reveal a cautiously positive orientation towards cultured meat, with attitudes and general acceptance exceeding the scale midpoint and two favourable psychographic segments—highly receptive consumers and cautious optimists—representing the majority of respondents. However, the link between meat-purchase behaviour and openness to cultured meat appeared relatively weak in this sample. Despite the modest positive associations observed between ethical and environmental motives, and the negative relationship between sensory-driven motives, the overall explanatory power of conventional meat-purchase motivations remained limited. The statistically significant yet modest structural congruence between behavioural and psychographic segmentation further corroborates the notion that the acceptance of cultured meat does not merely represent a straightforward extension of habitual meat-choice heuristics.

Instead, the propensity to accept cultured meat appears to be more strongly influenced by factors such as familiarity, perceived technological naturalness, ethical orientation and socio-demographic characteristics. It was evident that younger, urban and higher-educated consumers were more open to the concept, while technological risk and naturalness concerns remained significant barriers for those who were ambivalent. These findings underscore the necessity for targeted communication strategies that address informational gaps, clarify production processes and enhance trust in cellular-agriculture technologies. From a market perspective, early adoption is likely to be driven by consumers who already exhibit pro-innovation and sustainability-oriented values. However, broader acceptance will depend on transparent communication, regulatory clarity and sensory performance as products near commercialisation.

In view of the restricted explanatory capacity of the conventional meat-purchase motivations observed in this study, future research should incorporate additional psychosocial predictors that have demonstrated relevance in food technology acceptance contexts. These include trust in food governance and scientific institutions (which may moderate the technological concerns captured by TRNC), disgust sensitivity (consistently linked to rejection of novel protein sources in the literature), moralised eating orientations (e.g., ethical identity, moral conviction about animal agriculture), and broader food technology attitudes, such as general neophobia and perceived benefit-risk trade-offs.

Furthermore, the significant association between the willingness to substitute conventional meat with plant-based or insect-based alternatives and favourable cultured-meat orientations suggests that future studies should explicitly compare acceptance patterns across multiple alternative protein categories. These categories include plant-based proteins, hybrid meat products and precision fermentation-derived ingredients. This would allow an assessment to be made as to whether cultured meat acceptance reflects a broader ‘alternative protein openness’ portfolio or constitutes a distinct technology-specific response.

The present study makes an empirical contribution to the global cultured-meat literature by integrating behavioural and psychographic segmentation, thus providing stakeholders with actionable insights regarding the introduction of cultured meat in Central and Eastern Europe. This demonstrates that acceptance is multidimensional and cannot be inferred from conventional meat-purchasing patterns alone. It emphasises the importance of addressing technological perceptions, enhancing familiarity and recognising the heterogeneity of consumer responses in emerging protein markets.

## Figures and Tables

**Table 1:** Sociodemographic characteristics of the meat-eating sample (*n* = 425).

Demographic	Response Format	Number	Percentage
Gender	Female	281	66.1
	Male	144	33.9
Age	18–34 years old	178	41.9
	35–54 years old	176	41.1
	>54 years old	71	17.0
Education	Low level	43	10.1
	Medium level	69	16.2
	High level	313	73.7
Residence	Village	125	29.4
	City up to 100,000	90	21.2
	City of 100,000–500,000	71	16.7
	City over 500,000	139	32.7
Income	Low income (up to 5.000 PLN)	156	36.7
	Medium income (5.001–10.000 PLN)	174	41.0
	High income (above 10.000 PLN)	95	22.3
Status of having children	No	195	45.9
	Yes	230	54.1
Employment	No	136	32.0
	Yes	289	68.0

**Table 2:** Reliability and descriptive statistics of multi-item scales.

Scale	Items (*n*)	Cronbach’s α	McDonald’s ω	Mean	SD
Attitudes toward cultured meat (ATT_CM)	7	0.656	0.821	4.73	0.84
Intentions toward CM (INT_CM)	5	0.917	0.938	3.79	0.99
Technological risk and naturalness concern (TRNC)	5	0.821	0.878	3.19	0.91
General acceptance of cultured meat (GACM)	3	0.771	0.868	3.72	0.87

**Table 3:** Sociodemographic characteristics of meat-purchase motivation clusters.

Variable	Category	Cluster 1 High-Engagement, Quality-and-Values Driven	Cluster 2 Sensory-Focused, Moderately Engaged	Cluster 3 Low-Involvement, Indifferent
Gender	Female	160 (65.3%)	62 (25.3%)	23 (9.4%)
	Male	37 (27.8%)	76 (57.1%)	20 (15.0%)
Age group	18–34 years	98 (57.3%)	65 (38.0%)	8 (4.7%)
	35–54 years	86 (59.3%)	38 (26.2%)	21 (14.5%)
	55 and more	13 (21.0%)	35 (56.5%)	14 (22.6%)
Education level	Primary	3 (7.1%)	26 (61.9%)	13 (31.0%)
	Secondary	35 (54.7%)	22 (34.4%)	7 (10.9%)
	University	159 (58.5%)	90 (33.1%)	23 (8.5%)
Place of residence	City of 100–500 thousand	26 (40.0%)	28 (43.1%)	11 (16.9%)
	City over 500 thousand	69 (58.0%)	41 (34.5%)	9 (7.6%)
	City up to 100 thousand	49 (59.0%)	24 (28.9%)	10 (12.0%)
	Village	53 (47.7%)	45 (40.5%)	13 (11.7%)
Employment status	Not working	64 (49.2%)	50 (38.5%)	16 (12.3%)
	Working	133 (53.6%)	88 (35.5%)	27 (10.9%)
Parenthood	No	96 (51.6%)	77 (41.4%)	13 (7.0%)
	Yes	101 (52.6%)	61 (31.8%)	30 (15.6%)
Household income	Above 10,000 PLN	40 (48.8%)	32 (39.0%)	10 (12.2%)
	From 5000 to 10,000 PLN	91 (61.1%)	46 (30.9%)	12 (8.1%)
	Up to 5000 PLN	66 (44.9%)	60 (40.8%)	21 (14.3%)

**Table 4:** Sociodemographic and awareness characteristics of cultured-meat psychographic segments.

Variable	Category	Cluster 1 Highly Receptive Consumers	Cluster 2 Concerned Ambivalents	Cluster 3 Cautious Optimists	Cluster 4 Strong Sceptics
Previously heard of cultured meat	Not sure	6 (7.9%)	37 (48.7%)	31 (40.8%)	2 (2.6%)
	No	17 (14.0%)	50 (41.3%)	40 (33.1%)	14 (11.6%)
	Yes	83 (36.6%)	49 (21.6%)	84 (37.0%)	11 (4.8%)
Self-rated knowledge about cultured meat	Average	33 (35.1%)	22 (23.4%)	33 (35.1%)	6 (6.4%)
	No knowledge	25 (16.7%)	62 (41.3%)	49 (32.7%)	14 (9.3%)
	Poor	24 (16.4%)	48 (32.9%)	68 (46.6%)	6 (4.1%)
	Very well	6 (85.7%)	0 (0.0%)	1 (14.3%)	0 (0.0%)
	Well	18 (64.3%)	4 (14.3%)	4 (14.3%)	2 (7.1%)
Familiarity with production process	Not sure	32 (21.3%)	52 (34.7%)	60 (40.0%)	6 (4.0%)
	No	33 (16.7%)	70 (35.4%)	77 (38.9%)	18 (9.1%)
	Yes	41 (53.2%)	14 (18.2%)	18 (23.4%)	4 (5.2%)
Willingness to replace conventional meat with meat substitutes	No	10 (8.8%)	47 (41.2%)	34 (29.8%)	23 (20.2%)
	Yes, but not much.	36 (23.5%)	58 (37.9%)	56 (36.6%)	3 (2.0%)
	Yes	60 (38.0%)	31 (19.6%)	65 (41.1%)	2 (1.3%)
Gender	Female	75 (26.7%)	82 (29.2%)	104 (37.0%)	20 (7.1%)
	Male	31 (21.5%)	54 (37.5%)	51 (35.4%)	8 (5.6%)
Age group	18–34 years	52 (29.2%)	46 (25.8%)	70 (39.3%)	10 (5.6%)
	35–54 years	48 (27.3%)	45 (25.6%)	67 (38.1%)	16 (9.1%)
	55 years and more	6 (8.5%)	45 (63.4%)	18 (25.4%)	2 (2.8%)
Education level	Primary	1 (2.3%)	29 (67.4%)	9 (20.9%)	4 (9.3%)
	Secondary	12 (17.4%)	21 (30.4%)	26 (37.7%)	10 (14.5%)
	University	93 (29.7%)	86 (27.5%)	120 (38.3%)	14 (4.5%)
Place of residence	City of 100–500 thousand	13 (18.3%)	33 (46.5%)	21 (29.6%)	4 (5.6%)
	City over 500 thousand	36 (25.9%)	35 (25.2%)	53 (38.1%)	15 (10.8%)
	City up to 100 thousand	21 (23.3%)	32 (35.6%)	33 (36.7%)	4 (4.4%)
	Village	36 (28.8%)	36 (28.8%)	48 (38.4%)	5 (4.0%)
Employment status	Not working	25 (18.4%)	55 (40.4%)	49 (36.0%)	7 (5.1%)
	Working	81 (28.0%)	81 (28.0%)	106 (36.7%)	21 (7.3%)
Parenthood	No	51 (26.2%)	56 (28.7%)	78 (40.0%)	10 (5.1%)
	Yes	55 (23.9%)	80 (34.8%)	77 (33.5%)	18 (7.8%)
Household income	Above 10,000 PLN	28 (29.5%)	18 (18.9%)	39 (41.1%)	10 (10.5%)
	From 5000 to 10,000 PLN	52 (29.9%)	48 (27.6%)	65 (37.4%)	9 (5.2%)
	Up to 5000 PLN	26 (16.7%)	70 (44.9%)	51 (32.7%)	9 (5.8%)

## Data Availability

The dataset generated and analysed during the current study has been deposited in the Zenodo repository [98]. Do Conventional Meat-Purchase Motivations Predict Acceptance of Cultured Meat? A National Study Among Polish Consumer (Version 2). Zenodo. URL (23 January 2026) https://doi.org/10.5281/zenodo.18348457.

## Supplementary Materials

The following supporting information can be downloaded at: https://www.mdpi.com/article/10.3390/foods15040746/s1, Table S1. Overview of cultured meat: definition, benefits, limitations and regulatory status. Table S2. Survey questionnaire. Table S3. Scale Sources and Adaptation [96,97]. Table S4. Logistic regression model for cultured meat awareness.
