# Supplementary material for: Do Conventional Meat-Purchase Motivations Predict Acceptance of Cultured Meat? A National Study Among Polish Consumers
Source: Foods. 2026 Feb 18;15(4):746. doi: 10.3390/foods15040746 (PMC12939466; doi:10.3390/foods15040746)
Supplement: Supplementary file 1 [file foods-15-00746-s001.zip › Table S1.pdf]

Table S1. Overview of cultured meat: definition, benefits, limitations and regulatory status

Cultured meat is a new type of food produced from animal cells without killing the animals. It involves taking muscle stem cells from living animals and growing them in bioreactors. In this way, meat with similar nutritional value to traditional meat can be obtained without the need to breed and kill animals. Cultured meat has many potential benefits for humans, animals, and the planet. It can help reduce greenhouse gas emissions and save natural resources. It can also make meat products safer, reducing the risk of zoonotic disease transmission as well as contamination of meat with pathogens or residues of veterinary drugs and pesticides. In addition, it can be tailored to consumer preferences in terms of taste, texture, or nutritional value. It can provide an alternative for people who have stopped eating meat for ethical reasons. Limitations of this technology to date include high production costs, lack of regulation and lack of public acceptance. Cultured meat is currently the subject of intensive research in many countries around the world. Currently, cultured meat is approved in Singapore and Israel. In 2023, the USDA approved cultured chicken for sale, marking a significant milestone. In the European Union, cultured meat is classified as a "Novel Food" under EU Regulation 2015/2283. Pre-market authorization is required, but no applications have yet been submitted. A company called LabFarm has been established in Poland, and is working on chicken cultured meat production technology.
