# Supplementary material for: Do Conventional Meat-Purchase Motivations Predict Acceptance of Cultured Meat? A National Study Among Polish Consumers
Source: Foods. 2026 Feb 18;15(4):746. doi: 10.3390/foods15040746 (PMC12939466; doi:10.3390/foods15040746)
Supplement: Supplementary file 1 [file foods-15-00746-s001.zip › Table S2.pdf]

Table S2. Survey questionnaire

Number of question	Question/statement	Scale
Q1.	Have you heard of cultured meat?	Nominal scale (no, I'm not sure, yes)
Q2.	How would you rate your knowledge of cultured meat?	Nominal scale (no knowledge, poor, average, well, very well)
Q3.	Do you know how cultured meat is produced?	Nominal scale (no, I'm not sure, yes)
Q4.	Do you eat meat? If no please go to question #8	Nominal scale (no, yes)
Q5.	If yes, how often do you eat meat?	Nominal scale (very rarely, several time a month, once a week, several time a week, daily)
Q7.1.	What aspects are important to you when buying meat? [Price]	Likert scale (1—strongly disagree; 5—strongly agree)
Q7.2.	What aspects are important to you when buying meat? [Nutritional value]	Likert scale (1—strongly disagree; 5—strongly agree)
Q7.3.	What aspects are important to you when buying meat? [Caloricity]	Likert scale (1—strongly disagree; 5—strongly agree)
Q7.4.	What aspects are important to you when buying meat? [Taste]	Likert scale (1—strongly disagree; 5—strongly agree)
Q7.5.	What aspects are important to you when buying meat? [Appearance]	Likert scale (1—strongly disagree; 5—strongly agree)
Q7.6.	What aspects are important to you when buying meat? [Smell]	Likert scale (1—strongly disagree; 5—strongly agree)
Q7.7.	What aspects are important to you when buying meat? [Environmental impact]	Likert scale (1—strongly disagree; 5—strongly agree)
Q7.8.	What aspects are important to you when buying meat? [Animal welfare]	Likert scale (1—strongly disagree; 5—strongly agree)
Q9.	Would you be willing to replace meat with meat substitutes? (Plant proteins or insects)	Nominal scale (no, yes, but not much, yes)
Q10.	Cultured meat is safe	Likert scale (1—strongly disagree; 7—strongly agree)
Q11.	Cultured meat is healthy	Likert scale (1—strongly disagree; 7—strongly agree)
Q12.	Do you think cultured burgers will be as tasty as traditional burgers?	Likert scale (1—strongly disagree; 7—strongly agree)
Q13.	Eating cultured meat is disgusting.	Likert scale (1—strongly agree; 7—strongly disagree)
Q14.	Production of cultured meat will reduce consumption of natural resources.	Likert scale (1—strongly disagree; 7—strongly agree)
Q15.	The production of cultured meat will improve the welfare of livestock	Likert scale (1—strongly disagree; 7—strongly agree)
Q16.	Production of cultured meat will help feed the earth's growing population	Likert scale (1—strongly disagree; 7—strongly agree)
Q17.1.	Would you like to try cultured meat?	Likert scale (1—strongly disagree; 5—strongly agree)
Q17.2.	Would you choose to purchase cultured meat?	Likert scale (1—strongly disagree; 5—strongly agree)
Q17.3.	Would you be willing to pay more for cultured meat?	Likert scale (1—strongly disagree; 5—strongly agree)
Q17.4.	Would you be willing to prepare a meal for family or friends using cultured meat?	Likert scale (1—strongly disagree; 5—strongly agree)
Q17.5.	Would you be willing to try cultured meat if recommended by family or friends?	Likert scale (1—strongly disagree; 5—strongly agree)
Q18.1.	Eating cultured meat can be unsafe for health	Likert scale (1—strongly agree; 5—strongly disagree)

Q18.2.	I don't trust new foods	Likert scale (1—strongly agree; 5—strongly disagree)
Q18.3.	New technologies reduce the naturalness of food	Likert scale (1—strongly agree; 5—strongly disagree)
Q18.4.	Cultured meat is unnatural	Likert scale (1—strongly agree; 5—strongly disagree)
Q18.5.	I am concerned that eating cultured meat may have long-term negative health effects (no study or data available)	Likert scale (1—strongly agree; 5—strongly disagree)
Q19.	Is CM needed?	Likert scale (1—strongly disagree; 5—strongly agree)
Q20.	Is CM better than other meat alternatives?	Likert scale (1—strongly disagree; 5—strongly agree)
Q21.	Is CM in accordance with your standards?	Likert scale (1—strongly disagree; 5—strongly agree)
Q22.	Gender	Nominal scale (female, male)
Q23.	Age range (3 categories)	Nominal scale (18–34, 35–54, >55)
Q24.	Education level (3 categories)	Nominal scale (primary/vocational, secondary, university)
Q25.	Place of residence (4 categories)	Nominal scale (village, city up to 50 000, city from 50 000 to 100 000, city above 100 000)
Q26.	Employment status (2 categories)	Nominal scale (not working, working)
Q27.	Do you have children?	Nominal scale (no, yes)
Q28.	Monthly household income (3 categories)	Nominal scale (up to 5 000 PLN, from 5 000 to 10 000PLN , above 10 000PLN)
