# Supplementary material for: Do Conventional Meat-Purchase Motivations Predict Acceptance of Cultured Meat? A National Study Among Polish Consumers
Source: Foods. 2026 Feb 18;15(4):746. doi: 10.3390/foods15040746 (PMC12939466; doi:10.3390/foods15040746)
Supplement: Supplementary file 1 [file foods-15-00746-s001.zip › Table S3.pdf]

Table S3. Scale sources and adaptation

Scale	Items	Number of Items	Construct Description	Cronbach's α	McDonald's ω	Source / Adaptation
ATT_CM	Q10–Q16	7	Attitudes toward cultured meat (general evaluation, perceived appropriateness, appeal); Q13 reverse-coded; composite mean	0.656	0.821	Adapted from Bryant et al. (2019) [37], Siegrist & Hartmann (2020) [42]
INT_CM	Q17.1–Q17.5	5	Behavioural intention (willingness to try, purchase, and recommend cultured meat); composite mean	0.917	0.938	Adapted from Bryant et al. (2019) [37]
TRNC	Q18.1–Q18.5	5	Perceived technological risk and concerns about unnaturalness; all reverse-coded; higher = stronger concern; composite mean	0.821	0.878	Based on literature on technological neophobia (e.g., Siegrist, 2008 [96])
GACM	Q19–Q21	3	General acceptance of cultured meat (social need, perceived superiority, personal value alignment); composite mean	0.771	0.868	New scale developed for the purpose of this study based on Rogers (2003) [39]; Newton & Blaustein-Rejto (2021) [97]
Auxiliary Variables	Q1–Q3; Q7.1–Q7.8		Q1–Q3: Awareness (logistic regression DV); Q7.1-Q7.8: Meat motives (K-means input)	-	-	Steptoe et al. (1995)[45]; Graça et al. (2015)[47]
