# Supplementary material for: Do Conventional Meat-Purchase Motivations Predict Acceptance of Cultured Meat? A National Study Among Polish Consumers
Source: Foods. 2026 Feb 18;15(4):746. doi: 10.3390/foods15040746 (PMC12939466; doi:10.3390/foods15040746)
Supplement: Supplementary file 1 [file foods-15-00746-s001.zip › Table S4.pdf]

Table S4. Logistic regression model for cultured meat awareness.

Predictor	Coef.	SE	OR	OR 95% CI (lower)	OR 95% CI (upper)	p-value
Intercept	-1.412	0.635	0.244	0.070	0.846	0.026
C(Q22)[T.Male]	0.284	0.259	1.329	0.800	2.208	0.273
C(Q23)[T.35–54]	-0.294	0.321	0.746	0.397	1.399	0.360
C(Q23)[T.55 and more]	-1.653	0.405	0.191	0.087	0.423	0.000
C(Q24)[T.Secondary]	1.233	0.588	3.432	1.083	10.874	0.036
C(Q24)[T.University]	2.487	0.579	12.024	3.867	37.387	0.000
C(Q25)[T.City over 500,000 inhabitants]	0.740	0.369	2.097	1.017	4.323	0.045
C(Q25)[T.City up to 100,000 inhabitants]	-0.032	0.382	0.968	0.458	2.046	0.933
C(Q25)[T.Village]	0.059	0.367	1.060	0.516	2.177	0.873
C(Q26)[T.Working]	-0.739	0.323	0.478	0.253	0.901	0.022
C(Q27)[T.Yes]	0.080	0.277	1.083	0.629	1.864	0.773
C(Q28)[T.From 5000 to 10000 PLN]	0.088	0.287	1.092	0.622	1.918	0.759
C(Q28)[T.Up to 5000 PLN]	-0.134	0.322	0.875	0.465	1.645	0.678

*Reference categories: female; age 18–34 years; primary education; rural area; not working; no children; monthly household income > 10,000 PLN.
